# Identification and Ultrastructural Characterization of a Novel Nuclear Degradation Complex in Differentiating Lens Fiber Cells

**DOI:** 10.1371/journal.pone.0160785

**Published:** 2016-08-18

**Authors:** M. Joseph Costello, Lisa A. Brennan, Ashik Mohamed, Kurt O. Gilliland, Sönke Johnsen, Marc Kantorow

**Affiliations:** 1 Department of Cell Biology and Physiology, University of North Carolina, Chapel Hill, NC, United States of America; 2 Department of Biomedical Science, Florida Atlantic University, Boca Raton, FL, United States of America; 3 Ophthalmic Biophysics, L V Prasad Eye Institute, Hyderabad, India; 4 Biology Department, Duke University, Durham, NC, United States of America; University of Colorado Denver School of Medicine, UNITED STATES

## Abstract

An unresolved issue in structural biology is how the encapsulated lens removes membranous organelles to carry out its role as a transparent optical element. In this ultrastructural study, we establish a mechanism for nuclear elimination in the developing chick lens during the formation of the organelle-free zone. Day 12–15 chick embryo lenses were examined by high-resolution confocal light microscopy and thin section transmission electron microscopy (TEM) following fixation in 10% formalin and 4% paraformaldehyde, and then processing for confocal or TEM as described previously. Examination of developing fiber cells revealed normal nuclei with dispersed chromatin and clear nucleoli typical of cells in active ribosome production to support protein synthesis. Early signs of nuclear degradation were observed about 300 μm from the lens capsule in Day 15 lenses where the nuclei display irregular nuclear stain and prominent indentations that sometimes contained a previously undescribed macromolecular aggregate attached to the nuclear envelope. We have termed this novel structure the nuclear excisosome. This complex by confocal is closely adherent to the nuclear envelope and by TEM appears to degrade the outer leaflet of the nuclear envelope, then the inner leaflet up to 500 μm depth. The images suggest that the nuclear excisosome separates nuclear membrane proteins from lipids, which then form multilamellar assemblies that stain intensely in confocal and in TEM have 5 nm spacing consistent with pure lipid bilayers. The denuded nucleoplasm then degrades by condensation and loss of structure in the range 600 to 700 μm depth producing pyknotic nuclear remnants. None of these stages display any classic autophagic vesicles or lysosomes associated with nuclei. Uniquely, the origin of the nuclear excisosome is from filopodial-like projections of adjacent lens fiber cells that initially contact, and then appear to fuse with the outer nuclear membrane. These filopodial-like projections appear to be initiated with a clathrin-like coat and driven by an internal actin network. In summary, a specialized cellular organelle, the nuclear excisosome, generated in part by adjacent fiber cells degrades nuclei during fiber cell differentiation and maturation.

## Introduction

The unique embryonic development of the ocular lens provides a pattern of fiber cell formation and maturation that is present throughout life [1–3]. During the early stages of embryonic development in a typical vertebrate lens, the cuboidal cells lining the posterior of the lens vesicle elongate to fill the vesicle as primary fiber cells. The anterior cuboidal epithelium, under the influence of growth factors, such as fibroblast growth factor, leave the cell cycle at the lens equator and differentiate into new secondary fiber cells that elongate towards the lens poles to form the sutures [4–7]. Differentiation increases the expression of genes needed to produce large amounts of crystallin proteins unique to the lens that fill the expanding cell volume during the rapid elongation of fiber cells. These changes are accompanied by enlargement of the nucleus, reduction in condensed chromatin and development of prominent nucleoli, consistent with the enhanced production of cytoplasmic proteins. Shortly after fiber cell elongation and cytoplasmic filling, the differentiation program initiates the systematic removal of cellular organelles, especially endoplasmic reticulum (ER), mitochondria and nuclei, which may scatter light, to create and maintain a transparent organelle-free zone (OFZ) [8–12]. The OFZ was recognized in the late nineteenth century [13], as illustrated in a recent review [14], and the phenomenon has been studied for many decades. In early morphological studies, it was reported that just prior to the OFZ, nuclei were altered such that elongated nuclei became condensed, rounded and pyknotic [15–18]. Studies employing confocal microscopy of fluorescently labeled cellular components supported the pattern of nuclear degradation showing small nuclear remnants deep within the OFZ while other organelles appeared to form a sharp boundary at the OFZ [9]. Such findings suggest that multiple pathways may be involved, although the exact mechanisms leading to organelle loss are not well understood.

The nuclear changes suggest that apoptotic pathways might be activated without completing the process to cell death [14]. However, the nuclear condensation and breakdown processes are not consistent with apoptosis and executioner caspases typically activated in apoptosis do not appear to be involved in nuclear breakdown [19]. Other studies implicate a specific DNAse IIβ [20, 21], although DNase IIβ is thought to be a lysosomal enzyme that would require access to the nucleoplasm perhaps through the fusion of lysosomal vesicles near the OFZ [22]. Separate approaches have provided important evidence that ubiquitin-proteosome pathways may be involved in the degradation of nucleoplasm from within the nucleus [23]. Further studies suggested that ubiquitin pathways might regulate the nuclear degradation process by controlling the components involved in mitosis that is not carried to completion [24]. This hypothesis is supported by the study of a mouse model null for a cyclin-dependent kinase 1 (CDK1) that was found to be essential for nuclear degradation during the formation of the OFZ [25]. Recently, it was shown that autophagy was involved in the breakdown of fiber cell organelles other than the nucleus [26–28]. Subsequent important studies demonstrated that suppression of the MAPK/JNK pathway enhanced loss of lens organelles and formation of the OFZ, providing evidence that critical upstream autophagy signaling pathways mediate OFZ formation [29]. To date, no classical autophagic vesicles have been reported to contain detectable nuclear components in differentiating lens fiber cells raising the need for further ultrastructural analysis of nuclear degradation. Support for the hypothesis that nuclear breakdown by autophagy, or nucleophagy, occurs in mammalian cells comes from the study of granulosa cells of developing ovarian follicles and COS7 cells in culture, which displayed autophagic vesicles derived from the nuclear envelope [30]. In order to explore the unresolved mechanism of nuclear breakdown at the OFZ, we have employed confocal and transmission electron microscopy (TEM) ultrastructural methods that reveal key modifications to the nuclear envelope and associated structures during nuclear degradation in the chick embryo model used in numerous previous studies.

Details of membranous structures, including autophagic vesicles and the nuclear envelope, were exposed in high-resolution confocal images and thin section electron micrographs recorded from the capsule through the OFZ. Unlike mitochondria and other organelles that appear to be removed by autophagy during organelle degradation, as reported previously, no autophagic vesicles were associated with any stage of nuclear breakdown during the creation of the OFZ. Instead of autophagic vesicles associated with the nucleus, we observed the formation and action of a novel macromolecular complex that appears to be created by a cooperative process of adjacent fiber cells specifically to degrade the nuclear envelope. We have termed this complex the nuclear excisosome. Remarkably, the structural evidence suggests that the complex is, in part, composed of unique filopodial-like projections from adjacent cells that contact the outer nuclear envelope to initiate the breakdown process.

## Results

### A. Preservation of prominent features of classical fiber cells and autophagic vesicles in the region closest to the lens capsule

**Fig 1:**
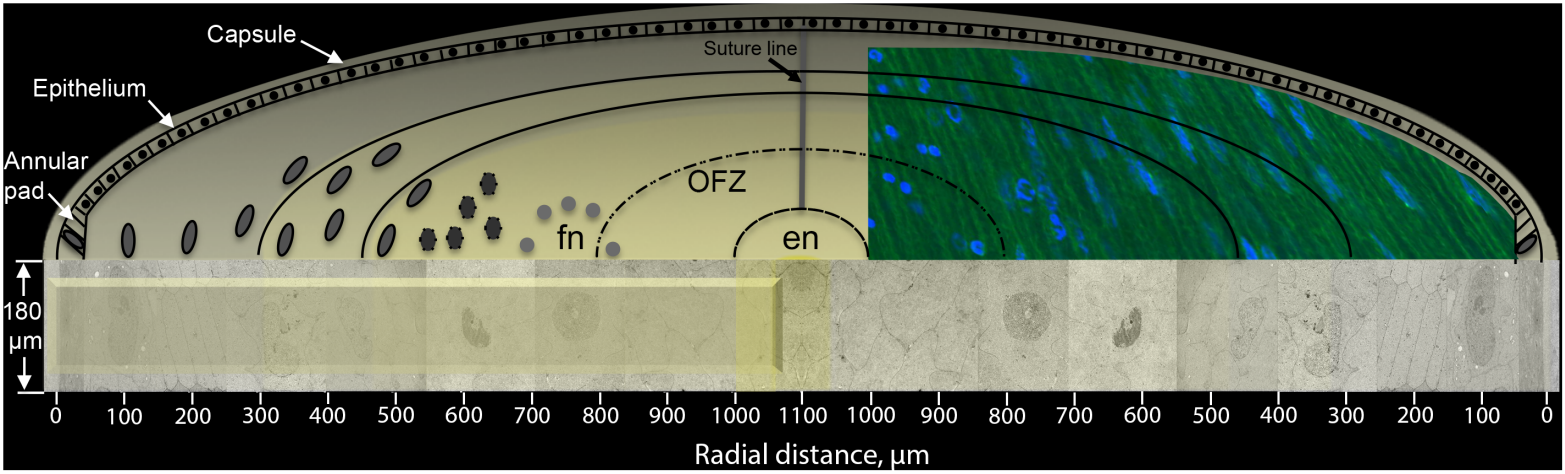
Schematic diagram of fiber cell cross-sections examined by thin section electron microscopy and longitudinal views by confocal microscopy. Regions sampled are from a typical Vibratome section of a Day 15 embryonic lens near the equatorial plane. Cell cross-sections are derived from electron micrographs at the depths indicated. The approximate position of the organelle-free zone (OFZ) for nuclei is indicated; for other membranous organelles, the OFZ is about 600 μm from the capsule. The mesa of the 180 μm model Vibratome section is raised for cutting 70 nm thin sections from the capsule through the fetal nucleus (fn) to the embryonic nucleus (en). Nuclei are depicted with an intact nuclear envelope (up to 300 μm depth), with modified nuclear envelopes up to 500 μm, with a disrupted nuclear envelope (500–700 μm depth) and without a nuclear envelope (>700 μm depth), roughly represented in the confocal inset. Several bands represent different regions of nuclear breakdown within the fetal nucleus (fn): the outer band (equivalent to the cortex in older lenses) is the region of classical fiber cell appearance, protein production and initiation of organelle degradation by autophagy; the second band represents the formation of the nuclear excisosome and the beginning of nuclear degradation; the next band up to the OFZ represents the breakdown of the nuclear envelope and formation of pyknotic nuclear fragments. Not drawn to scale.

Fiber cell cross-sections are examined near the equatorial plane in Day 12–15 lenses from the capsule to the embryonic nucleus with emphasis given to images from Day 15 lenses (Fig 1). In the outermost layer up to about 100 μm depth from the capsule, the capsule, annular pad and outer classical fiber cells are found with excellent preservation of cellular details. In this region, many autophagic vesicles are also observed. Modifications to the nuclear envelope are found mainly in the next region about 300–500 μm from the capsule. The final stages of nuclear breakdown and formation of the OFZ are found in the inner region about 500–800 μm depth. Nuclear changes were recorded in confocal images. Within a single 70 nm thin section from a mesa extending from the capsule through the OFZ, ultrastructural details are clearly visible revealing discrete stages of nuclear breakdown and the key cellular structures that facilitate the process.

**Fig 2:**
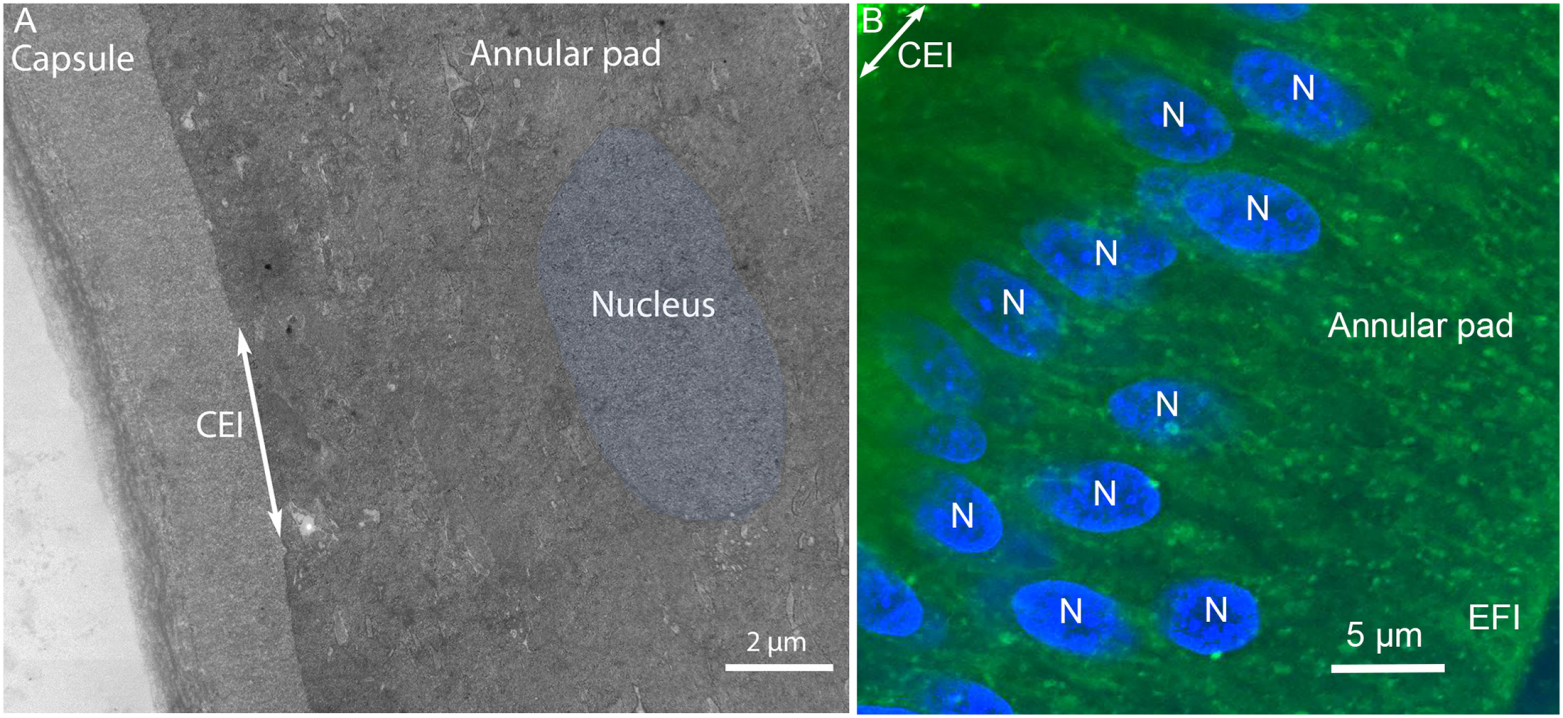
Overview of the capsule and annular pad. (A) Electron micrograph. (B) Confocal image. The capsule, annular pad and the capsule-epithelial-interface (CEI; double arrow) are indicated. One nucleus (Fig 2A, blue) is highlighted because it is difficult to visualize among the densely stained epithelial cells of the annular pad. Numerous nuclei are stained in the confocal image of an equivalent region of the annular pad (Fig 2B, N). Other membranous organelles such as lysosomes, mitochondria and endoplasmic reticulum are visible as globular objects but not distinguished. EFI is the epithelial to fiber cell interface.

The interface between the capsule and anterior epithelium is smooth and uniform (Fig 2). The annular pad epithelium has variable thickness in this region and stains densely close to the equatorial plane where some organelles can be identified, such as a prominent nucleus (Fig 2A, blue) and stained nuclei in confocal images (Fig 2B, N). The capsule-epithelium interface (CEI) is used here as a reference plane and all images are captured with a measured radial distance from the CEI. This distance is of course relevant only to Day 15 lenses, because the rapid growth in this embryonic stage gives a pronounced increase in diameter each day.

### B. Laser scanning confocal imaging of longitudinally oriented fiber cells

**Fig 3:**
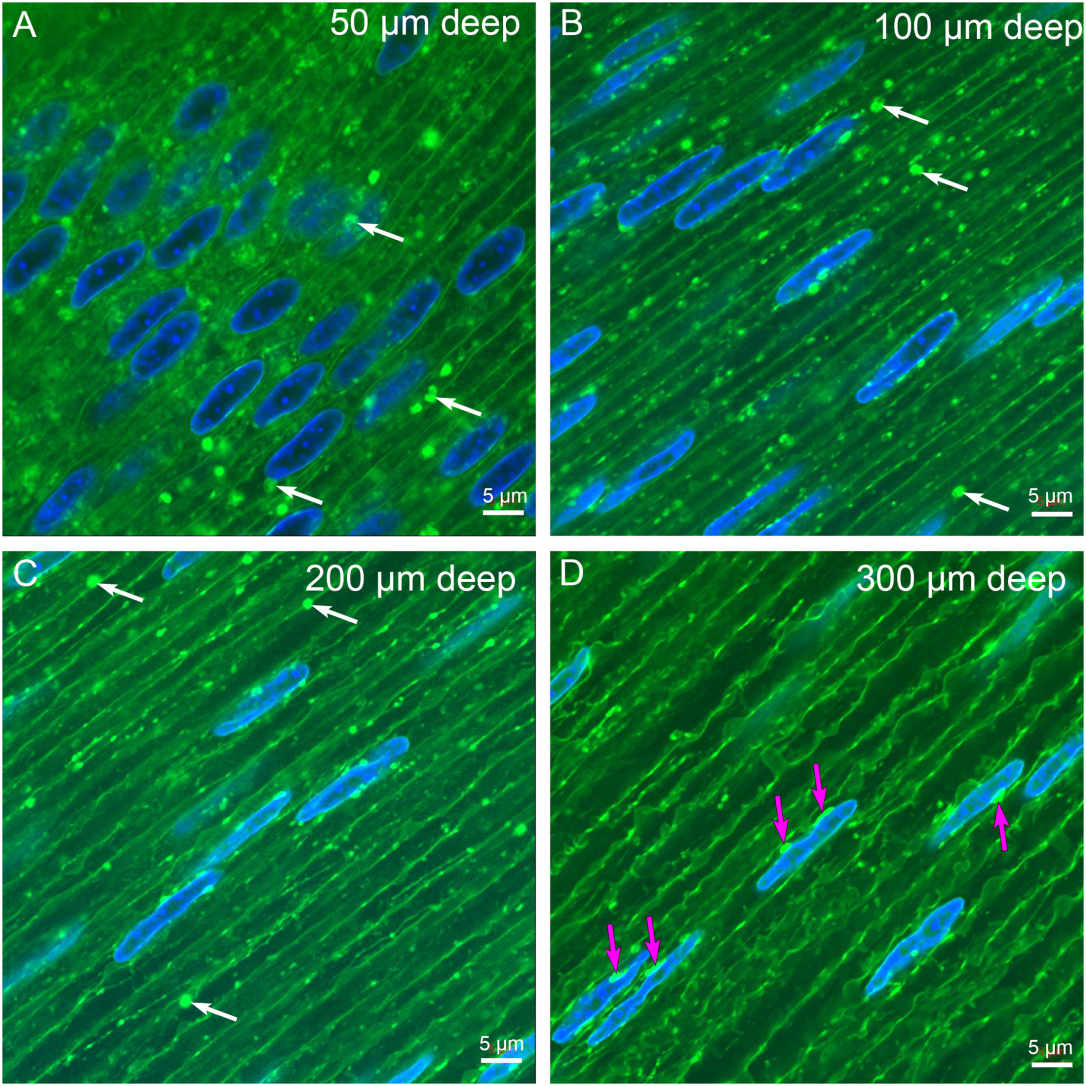
Laser scanning confocal imaging of D15 Vibratome sections. The depth from the capsule-epithelium-interface is indicated in the upper right corner of each image here and in subsequent images. (A) At 50 μm depth, the nuclei are large, oval and active indicated by the deep 4’,6-Diamidino-2-Phenylindole (DAPI) staining. The cells have uniform parallel borders except where nuclei distort the cellular shape. The membranes stained with 1,1’-Dioctadecyl-3,3,3’,3’-Tetramethylindocarbocyanine Perchlorate (DiI) are uniform in staining and smooth in topology. Numerous vesicular structures are visible that represent membranous organelles including mitochondria, lysosomes, endoplasmic reticulum and Golgi, as well as large circular objects that may represent autophagic vesicles (arrows). (B) At 100 μm depth, the intensely staining vesicular structures (arrows) predominate the background around nuclei that appear to be smaller in average diameter and lighter staining. (C) At 200 μm depth, a few large vesicular structures are visible (arrows), although the overall number of labeled structures is greatly reduced at the outer edge of the organelle-free zone. The nuclei are thin overall and sometimes irregular in width. (D) At 300 μm depth, the nuclei are thin, irregular in width and decorated with oblong brightly staining objects that appear to be attached to the DiI-stained nuclear envelope (magenta arrows). These appear prominent in part because they are not circular and there are very few vesicular structures visible. These structures are the first clear indication that there is a distinct complex that appears to be modifying the nuclei at the edge of the organelle-free zone. Note that the fiber cells are large in diameter and irregular in shape consistent with their depth from the capsule.

High resolution confocal images reveal important and unique features of fiber cell architecture and contents at specific distances from the capsule (Fig 3). The fiber cells closest to the capsule (Fig 3A) have large bulbous nuclei with intense DAPI staining located within cells of uniform width and smooth membrane topology. Numerous vesicular structures are found in this region consistent with the presence of organelles needed for protein production, as well as large circular structures with irregular staining that may represent autophagic vesicles (Fig 3A, arrows). At a depth of about 100 μm, the cellular structure is significantly different with nuclei that are thinner and lighter staining and with cells that contain few vesicular structures (Fig 3B). Some of the remaining vesicular structures are large and similar to those found in the outer layers (Fig 3B, arrows). At a depth of 200 μm, the fiber cells contain nuclei that are narrow and irregular in diameter and lightly stained (Fig 3C). The number of cytoplasmic vesicles is clearly lower, consistent with loss of organelles near the OFZ and the decrease of about a factor of five of autophagic vesicles to a depth of 250 μm for D12 chick embryos reported previously [27]. This decrease is significant because there are relatively few vesicular structures by 300 μm depth (Fig 3D). Nuclei in this region display pronounced elongated structures associated with the nuclear envelope (Fig 3D, arrows). The nuclear envelope takes up the fluorescent dye as well, appearing as a thin coat around the DAPI stained nuclei, whereas the labeled objects are significantly brighter than the nuclear envelope or the adjacent plasma membranes. This distinctive staining indicates that the labeled objects take up and concentrate the lipid dye specifically to highlight new structures that have not been reported previously.

**Fig 4:**
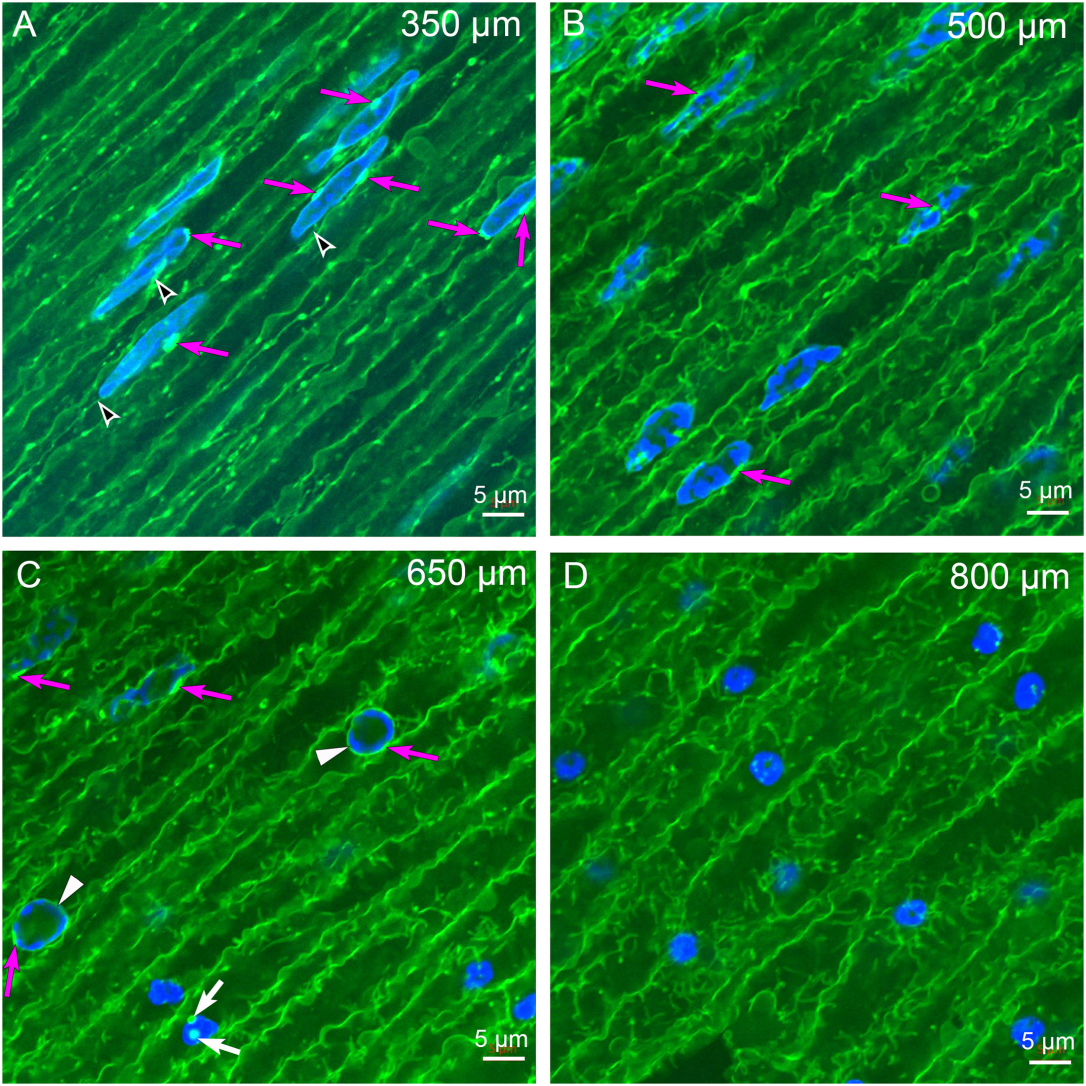
Laser scanning confocal imaging near the organelle-free zone. (A) At 350 μm depth, the cell shape is irregular and very few vesicular organelles are present within cells. Several examples of the brightly labeled structures located at the nuclear envelope are indicated (magenta arrows), as are potential links to the plasma membrane (black arrowheads). Both these structures can be better appreciated in z-series optical sections (see S1 Fig). (B) At 500 μm depth, the nuclei are irregular in shape and are beginning to degrade. Some nuclei have bright staining close to the nuclear envelope (magenta arrows) while others do not. (C) At 650 μm depth, the cells are larger and more irregular in shape. The nuclei are disrupted with two showing objects associated with the nuclear envelope (magenta arrows, upper left). The two examples in the center have circular shapes, faint staining of associated structures (magenta arrows) and light staining of the nuclear envelope (white arrowheads). These appear to be in the final stages of breakdown of the nuclear envelope as the four examples on the lower right are small globular remnants of nuclei with no visible nuclear envelope. Two small circular dots (white arrows) may be remnants of nuclear envelope breakdown but are not consistent with the vesicular structures in previous images. (D) At 800 μm depth, the cells are very large and irregular with several globular remnants of nuclear breakdown stained brightly with 4,6-diamidino-2-phenylindole (DAPI). These are most likely the pyknotic nuclei described in the early literature.

At a slightly greater depth, each thin, irregular nucleus has at least one and some have several bright oval structures associated with the nuclear envelope (Fig 4A, arrows). Some fine structures appear to connect the nuclei with adjacent plasma membrane (Fig 4A, arrowheads), although the details of this relationship will become clearer in subsequent higher resolution TEM images. The unique bright structures associated with the nuclear envelope and some potential intracellular connecting strands can be followed in z-series optical sections (S1 Fig) revealing that the objects along the z-axis are about 1–2 μm thick. It should be emphasized that, although these confocal images are recorded in Super Resolution mode of a Zeiss LSM 880 Airyscan confocal laser scanning microscope (LSM) with x,y-plane resolution of about 140 nm, the details of the internal structures of the bright staining objects and possible associated processes cannot be resolved at this resolution. However, the overall modifications of the nuclei at the OFZ are clearly evident. At 500 μm depth, the nuclear shape and staining are very irregular with only a few bright structures visible (Fig 4B, arrows). At 650 μm depth, a transition in nuclear shape is evident with the irregular nuclei sometimes appearing as smaller circular objects with a well-stained perimeter, which is most likely the remnants of the nuclear envelope (Fig 4C, arrowheads). Some nuclei in this region are condensed further to small circular objects, sometimes displaying the lipid dye, (Fig 4C, white arrows) that are distinct from vesicles in the outer layers or the oblong bright structures in the same image. By a depth of 800 μm, the only nuclear objects observed are small spherical remnants (Fig 4D) that represent the pyknotic nuclei reported in the literature [9]. Note that the fiber cells in this region are large and their shape is very complex.

### C. Classical fiber cells closest to the lens capsule revealed by thin section transmission electron microscopy

**Fig 5:**
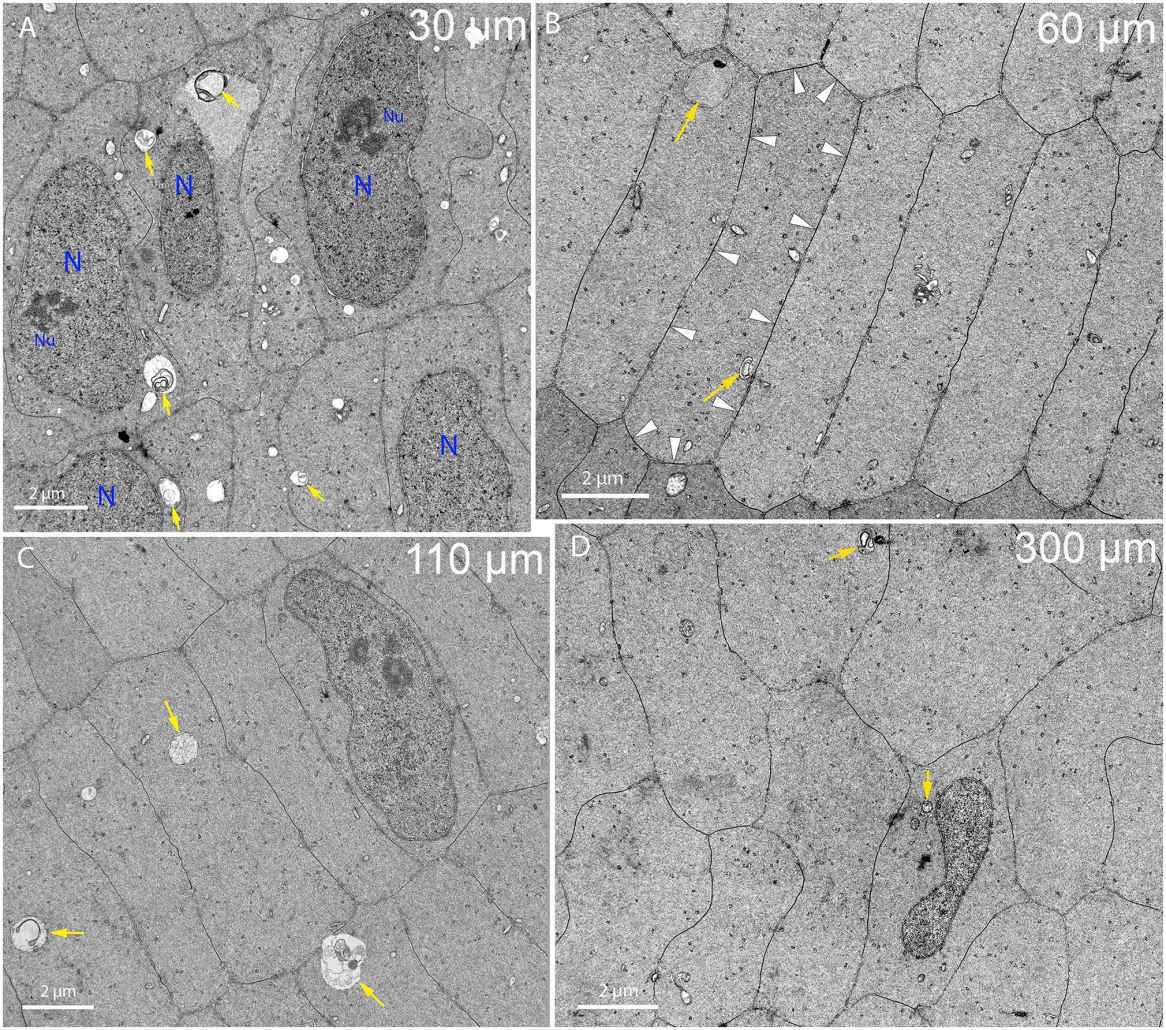
Fiber cell ultrastructure near the bow region up to a depth of 300 μm. (A) Nuclei of the bow region are present among well-defined radial cell columns. Many autophagic vesicles (arrows) are visible as well as numerous other smaller organelles. Two nuclei (N) have clear nucleoli (Nu). Day 15 lens imaged 30 μm from the capsule-fiber cell interface. (B) Just beyond the nuclei of the bow region, classical fiber cells with hexagonal 2 μm x 10 μm cross-sections in radial cell columns are observed. Autophagic vesicles (arrows) are present as are other organelles where protein production is still occurring. The dark stained globular clusters in the cytoplasm are probably polysomes. Extensive gap junctions that appear as thin dark lines on broad and narrow faces cover about 50% of the cell surface as indicated for one cell (arrowheads). Day 15 lens about 60 μm from the capsule-fiber cell interface. (C) Fiber cells just outside the organelle-free zone (OFZ) appear normal. Note the nucleus is slightly irregular in shape but otherwise normal in appearance with portions of a nucleolus visible. Autophagosomes with double membranes are indicated (yellow arrows), although they decrease in number corresponding to a decrease in membranous organelles in the cytoplasm through this region. The fiber cells have hexagonal shapes, which are somewhat irregular in size and slightly more rounded. Day 15 lens about 110 μm from the capsule-fiber cell interface. (D) Nuclei at the edge of the OFZ display irregular shapes. Note the pronounced indentation and thin region, which if projected along the nuclear length would suggest a significant reduction in nuclear volume. Irregular nuclear shapes including indentations have been reported previously [29]. Autophagic vesicles (arrows) and other organelles including mitochondria and endoplasmic reticulum are also present although quite rare. An important feature of this region is the irregular shape and rounded edges of the fiber cells. Day 15 lens about 300 μm from the capsule-fiber cell interface.

Just beneath the annular pad at about 30 μm depth, the appearance of radial cell columns of flattened hexagonal fiber cells cut in cross-section is obvious, as are several nuclei in this region from the bow zone (Fig 5A). Note the numerous autophagic vesicles (Fig 5A, arrows) supporting their previously reported involvement in the degradation of membranous organelles [27]. Many are double membrane structures consistent with their definition as autophagosomes. All observed nuclei have similar features, including a preserved nuclear envelope, numerous nuclear pores (small black dots) and granular nucleoplasm, which is easily distinguished from the adjacent cytoplasm. Two of the nuclei display sections through nucleoli. Because these elongated nuclei are cut in cross-section, nucleoli in other nuclei are out of the plane of section and nuclear shapes will change as the section nears the tapered ends of nuclei. Just deeper to this region, at about 60 μm from the CEI where nuclei are not in the field of view (Fig 5B), the radial cell columns with sharp contours of the flattened hexagons, sparse organelles and uniform cytoplasm are indications of well-preserved fiber cells outside the OFZ. At about 110 μm from the CEI, intact normal nuclei can still be detected (Fig 5C) along with autophagic vesicles (including some with double membranes) and slight variations in cell shape. This region is still clearly outside the OFZ. Near to 300 μm depth, the nuclei and cell shape changes are more evident. Radial cell columns are more difficult to define and cells are both more irregular in cross-sectional area and more rounded (Fig 5D). Most important is that the nuclei begin to display pronounced indentations (Fig 5D). Because this view is a cross-section of a nucleus, the indentation, if extended all along the nucleus, would indicate a major change in nuclear volume. However, the nuclear envelope is uniform without blebs or wrinkles, indicating that the surface area has adjusted to changes in internal nuclear volume. Although there are a few organelles observed adjacent to the nucleus, such as mitochondria, there is no indication of disruption of the nuclear envelope or loss of nuclear mass other than the indentation. This change in nuclear shape is consistent with the nuclei in confocal images (Fig 3).

### D. Breakdown of the nuclear envelope and modification of the nucleoplasm 300–500 μm from the capsule

**Fig 6:**
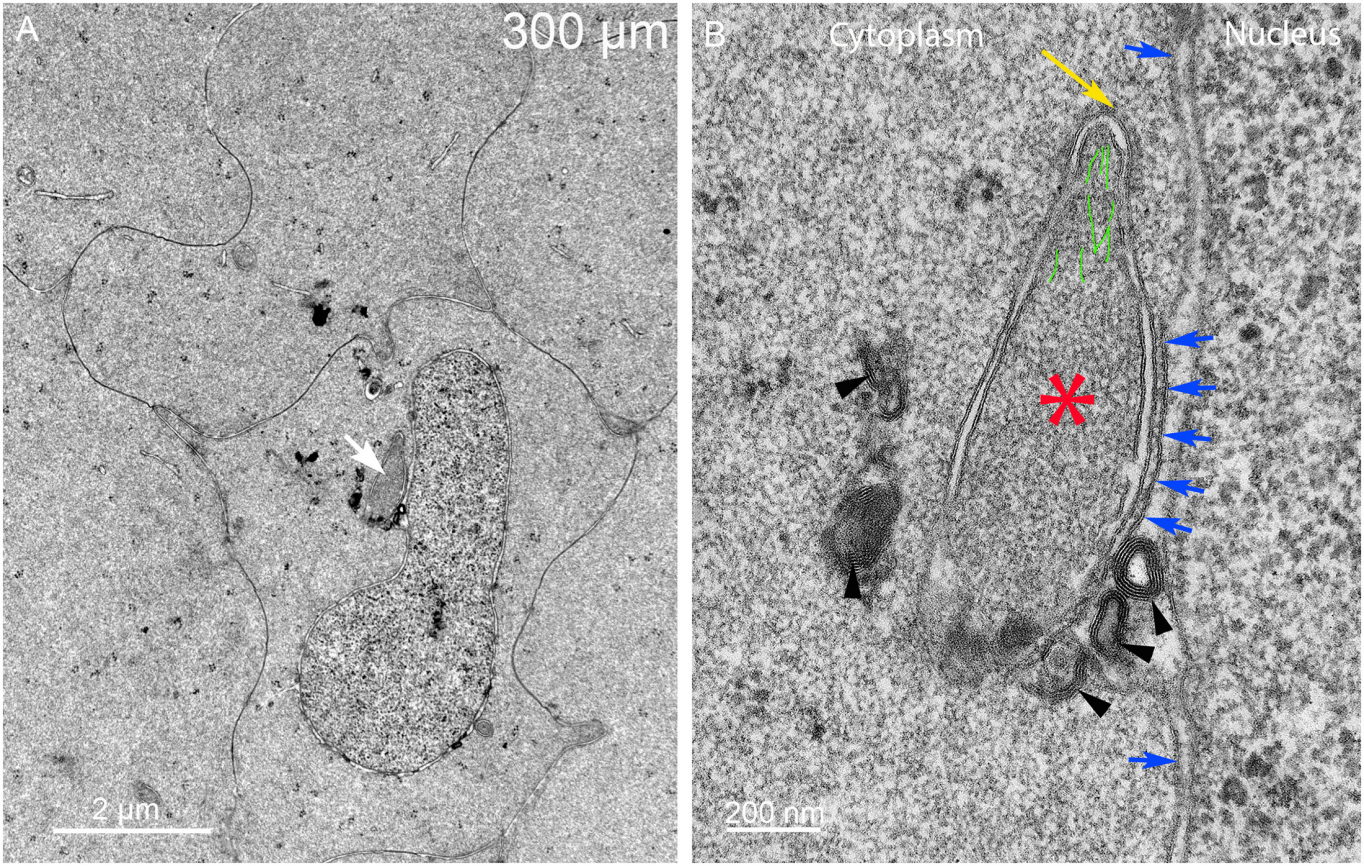
Indentations often reveal a large macromolecular complex adherent to the nuclear envelope. (A) Low magnification shows a complex (arrow) that has an unusual asymmetric shape and irregular densities associated with one surface. (B) High magnification is necessary to identify the components of the complex. The core (asterisk) has a uniform texture similar to the adjacent cytoplasm (distinct from nucleoplasm, lysosomes or any type of autophagosome) and is surrounded mainly by two membranes. The outer membrane is closely adhered to the outer nuclear envelope and distorting that membrane (five central blue arrows). The other segments of the outer nuclear membrane (remaining blue arrows) have a normal association with the inner nuclear membrane. The tip is narrowed and covered by a protein layer (yellow arrow) that is 25 nm thick with repeating protein densities that are similar to clathrin and its adapter proteins. The interior of the tip contains fibers (green lines) that are similar actin microfilaments often seen in lens epithelium, fiber cell cytoplasm and elongating ball-and-socket devices. The dense bodies (arrowheads) are multilayered bilayers with 5 nm spacing typical of pure lipid (without integral proteins). Day 15 lens 300 μm from the capsule-fiber cell interface.

Nuclei near the 300 μm depth sometimes reveal unique structures associated with the nuclear envelope (Fig 6). The nuclear shape is narrow on one end where a large structure is adjacent to the outer nuclear membrane (Fig 6A, arrow). At higher magnification, it is clear that the structure is a macromolecular complex with numerous components (Fig 6B). The arrangement of components is different from any organelle previously described in lens fiber cells from any source. The core of the complex is a uniformly dense material that is similar to the adjacent cytoplasm (Fig 6B, asterisk) nearly surrounded by two layers of membranes. At first glance, this pattern is suggestive of a double membrane autophagosome; however, close examination reveals that the core is uniform (inconsistent with any form of autophagosome) and one surface is closely opposed to the nuclear envelope. Furthermore, the complex is extremely asymmetric with unique ends. The broad end is associated with numerous multilamellar assemblies (Fig 6B, arrowheads) and the narrow end is tapered to a rounded point covered by a distinctive layer (Fig 6B, yellow arrow). This surface layer is about 25 nm thick with repeating spikes, remarkably similar to the clathrin coat and adapter proteins observed during the formation of fiber cell ball-and-socket interlocking devices [31]. The interior of the rounded end appears to contain filaments (Fig 6B, green lines; also see S2 Fig) that are similar to the actin microfilaments within protrusions that develop into ball-and-sockets [31]. A most unusual feature is the close association of the complex specifically with the outer membrane of the nuclear envelope (Fig 6B, blue arrows), which appears to be pulled away from its normal association with the inner nuclear envelope. This macromolecular complex is most likely equivalent to the bright fluorescent objects seen adjacent to the nuclear envelope in confocal images where the dye is concentrated in the lipid-rich multilamellar objects.

**Fig 7:**
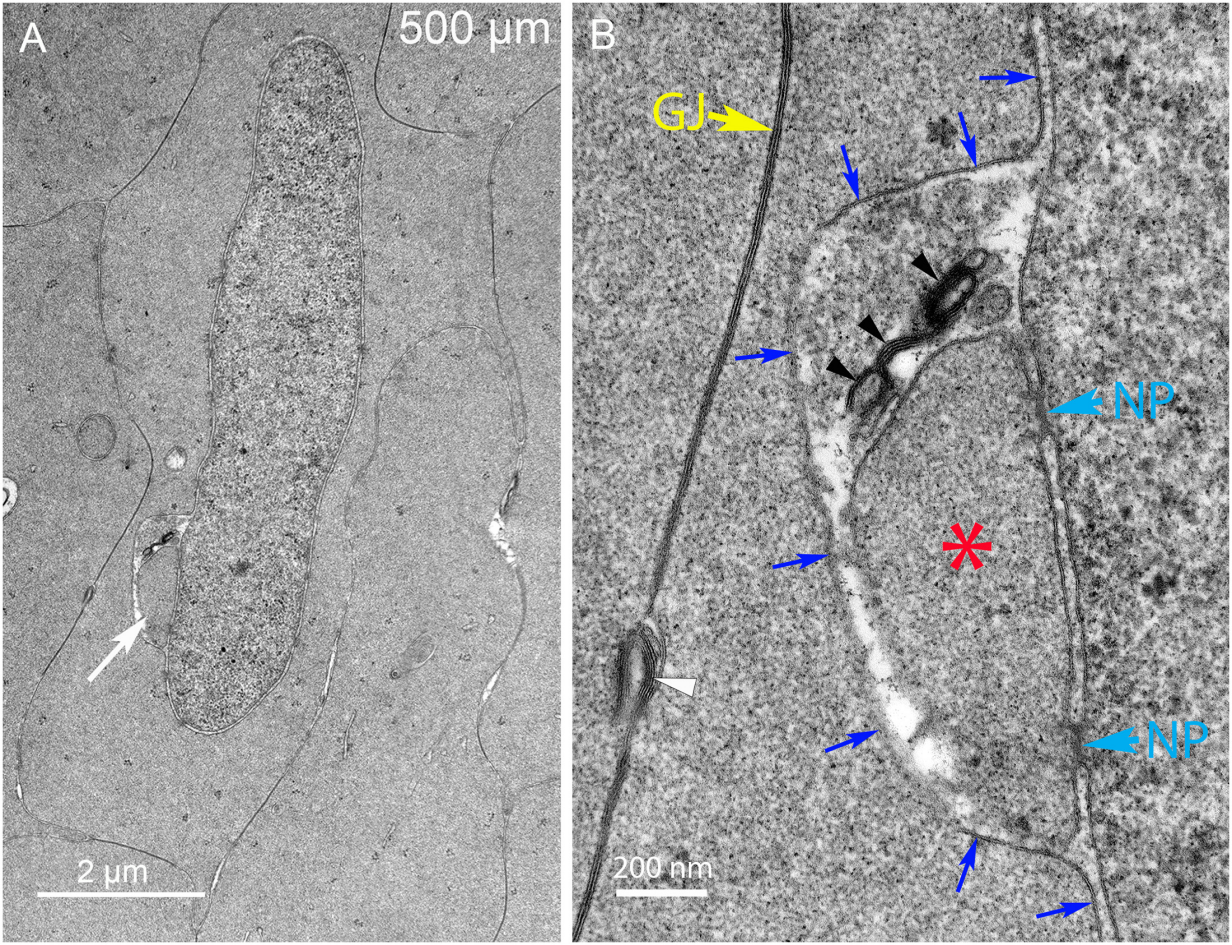
A distinct form of the degradation complex appears to attack the inner nuclear membrane. (A) Low magnification shows a large oblong complex adjacent to the nucleus (arrow). (B) High magnification shows that the complex is nearly completely contained under the outer nuclear membrane (blue arrows). The core (asterisk) is uniform in texture and similar to the adjacent cytoplasm. The core is associated with the inner nuclear membrane and two nuclear pores (NP); however, the position of the multilamellar material suggests that the inner nuclear membrane is being modified. There is no conical tip or clathrin-like coat. A typical gap junction (GJ) is indicated, as is a cluster of thin multilamellar membranes (white arrowhead) associated with the plasma membrane. Day 15 lens about 500 μm from the capsule-fiber cell interface.

A separate and distinct configuration of components is observed in some degradation complexes in which the core comes in close contact with the inner nuclear membrane. Such a complicated topological pattern is illustrated in the complex associated with a different nucleus near 500 μm depth (Fig 7). At low magnification, the large complex appears to be an appendage attached to the nucleus (Fig 7A, arrow) whereas at high magnification the complex is found to be nearly completely contained within the nuclear envelope perinuclear cisternal space (Fig 7B). The inner nuclear envelope membrane is intact with two nuclear pores indicated (Fig 7B, NP) and the outer membrane of the nuclear envelope bulges out to cover the complex (Fig 7B, blue arrows). The core is again similar in texture to the adjacent cytoplasm and thus is not an autophagosome or lysosome (Fig 7B, asterisk). Also the ends are different from the previous example with no indication of a clathrin-type coat and the multilamellar objects are clearly within the nuclear envelope cisternal space. We hypothesize that the first form contacts and degrades the outer nuclear membrane followed by fusion of the contact zone to move the core into the nuclear cisternal space to contact and degrade the inner nuclear membrane. However, fusion intermediates to validate this hypothesis have not been found, thus the proposal remains a hypothesis. Common features between these forms are the close association with the nuclear envelope outer surface, the similar core structures and the multilamellar objects. The multilamellar objects have high curvatures and narrow repeat spacing of 5 nm consistent with lipid-rich bilayers (without integral protein) and thus the assemblies are hypothesized to degrade the nuclear envelope by separating nuclear membrane proteins and lipid to eventually recycle usable lipid components and degrade protein components. Two of the multilamellar objects in the first form (Fig 6B) appear to be within the cytoplasm and not connected to the complex, suggesting that they may be diffusing toward the plasma membrane where similar multilamellar aggregates are found (Fig 7B, white arrowhead; also see S3 Fig). Numerous examples of such complexes have been recorded in this region in Day 15 lenses and equivalent structures have been observed in Day 13 and Day 14 lenses. Additional examples illustrate the range of morphologies of these unique complexes (S3 Fig).

**Fig 8:**
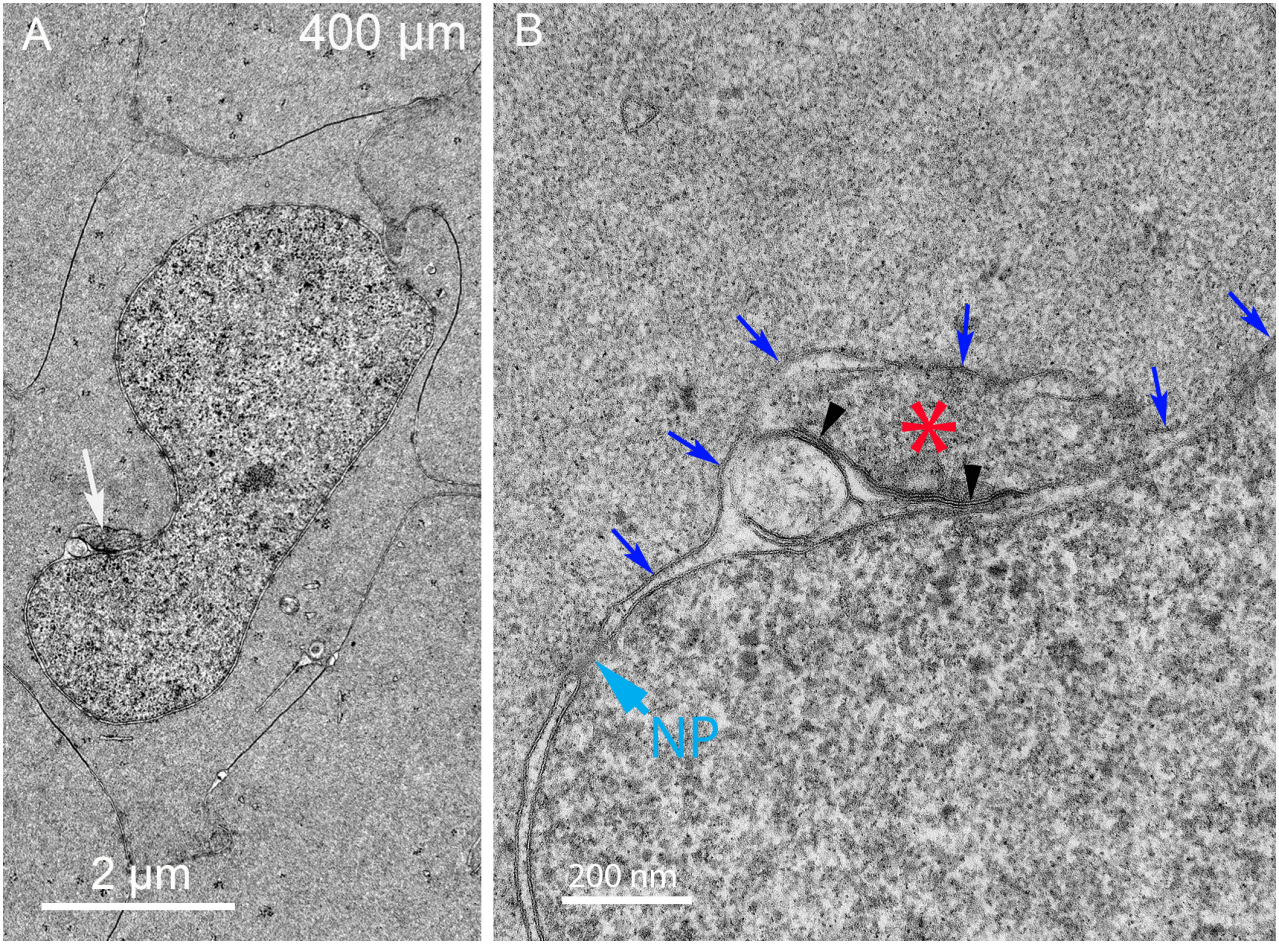
Complexes have been observed associated with a small number of thin multilamellar membranes within an indentation. (A) Low magnification shows a complex (arrow) in an indentation with variable densities. (B) High magnification shows that the complex is beneath the outer nuclear membrane (blue arrows) and the presumed core (asterisk) stains darkly with similar texture to the adjacent cytoplasm. A lighter staining circular region of unknown origin bridges the nuclear envelope with a small accumulation of multilamellar membranes. The molecular events within the complex are unknown, although the proximity of the domains is consistent with breakdown of the inner nuclear membrane. An intact nuclear pore (NP) appears not to be disturbed by the presence of the complex. Day 15 lens 400 μm from the capsule-fiber cell interface.

**Fig 9:**
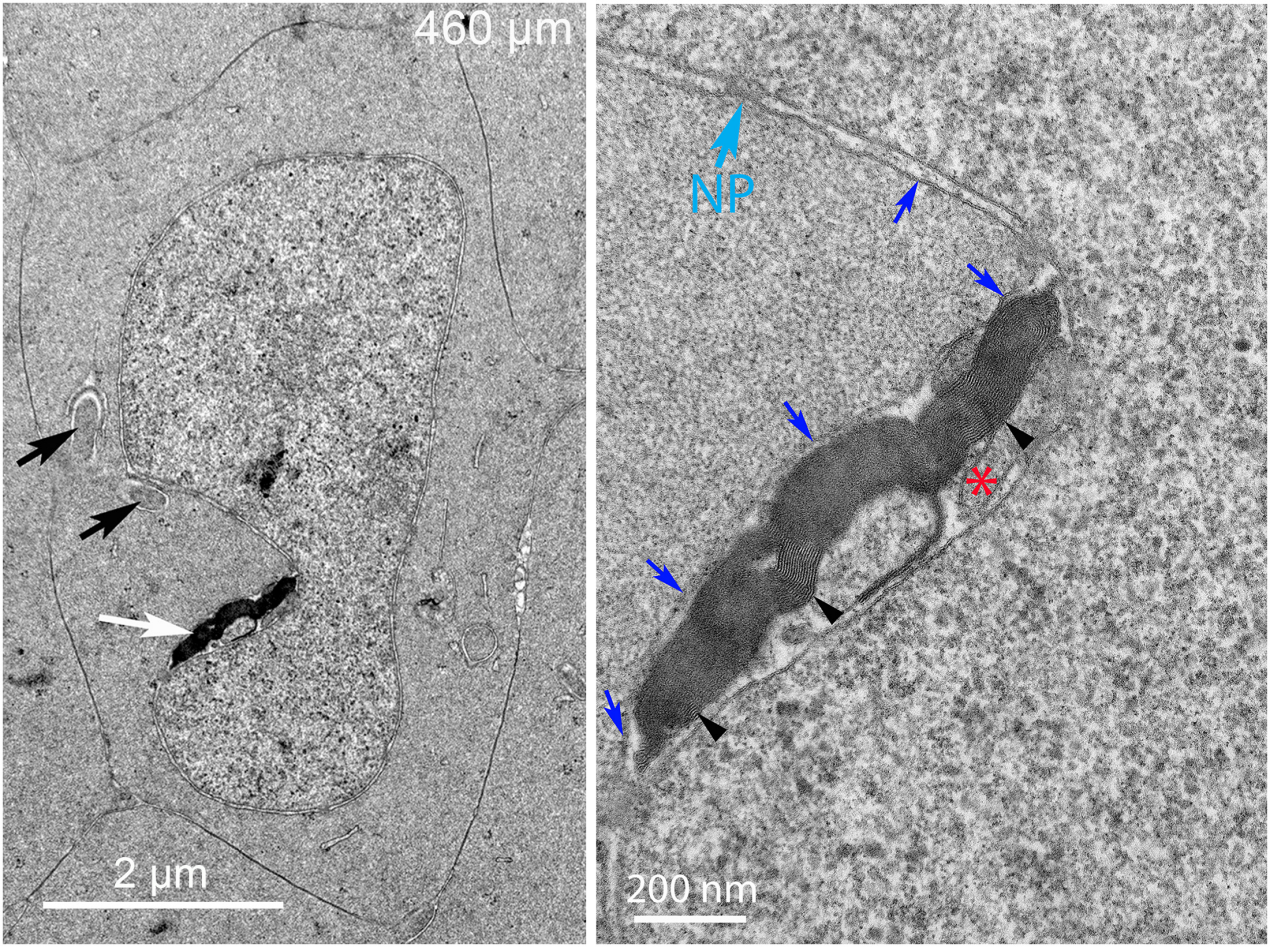
Many complexes contain large aggregates of thin multilamellar membranes. (A) Low magnification shows a large complex in a nuclear indentation with an intensely stained aggregate within the complex (white arrow). In addition, there are two extended objects adjacent to the nucleus (black arrows) that are early stages of complex formation as discussed below. (B) High magnification shows that the large dark object is an extensive collection of thin bilayer membranes (arrowheads) that most likely represent pure lipid derived from the breakdown of the nuclear envelope. Bilayer average spacing was 5.2 nm (n = 59). The complex is contained beneath the outer nuclear membrane (blue arrows). The presumed core (asterisk) is small but similar in texture to the adjacent cytoplasm and not to autophagosomes or lysosomes. An intact nuclear pore (NP) is indicated. Day 15 lens 460 μm from the capsule-fiber cell interface.

In another example, a nucleus with a pronounced indentation displays a prominent complex within the indentation (Fig 8). It is not possible at low magnification to determine the geometry of the complex (Fig 8A, arrow), while at high magnification it is clear that the complex is contained within the nuclear envelope space and the outer membrane of the nuclear envelope bulges around the complex, as in the previous example (Fig 8B, blue arrows). The core is uniform in density and the multilamellar regions of 5 nm spacing are sparse (Fig 8B, arrowheads). An additional example of a nucleus with a similar indentation shows a complex within the indentation that is very pronounced (Fig 9A, white arrow) and distinct from two other membrane bound projections that will provide clues about the origin of the complexes (Fig 9A, black arrows). At high magnification, the intensely staining portion of the large complex is due to a massive collection of multilamellar bilayers with 5 nm spacing (Fig 9B, arrowheads). This structure is adjacent to a core (Fig 9B, asterisk) and contained beneath an outer nuclear envelope membrane as in the two previous examples (Fig 9B, blue arrows). Such a large volume of thin lipid multilayers is consistent with the removal of lipid from the nuclear envelope, because the nucleus is the only membranous organelle in this region with sufficient lipid to serve as source of the multilamellar structure. Together these structural complexes (including S3 Fig) constitute the degradation complex that we have termed the “nuclear excisosome” specifically formed to degrade the nuclear envelope. All forms of the nuclear excisosome observed thus far have associated multilamellar lipid-rich aggregates that would preferentially concentrate lipid dyes such as DiI used in the confocal experiments. Thus, the evidence suggests that the brightly fluorescent structures attached to the nuclear envelope in confocal images (Figs 3D and 4A; S1 Fig) represent nuclear excisosomes. Further evidence is needed to determine the origin of the nuclear excisosome.

### E. Possible origin of the nuclear excisosome

**Fig 10:**
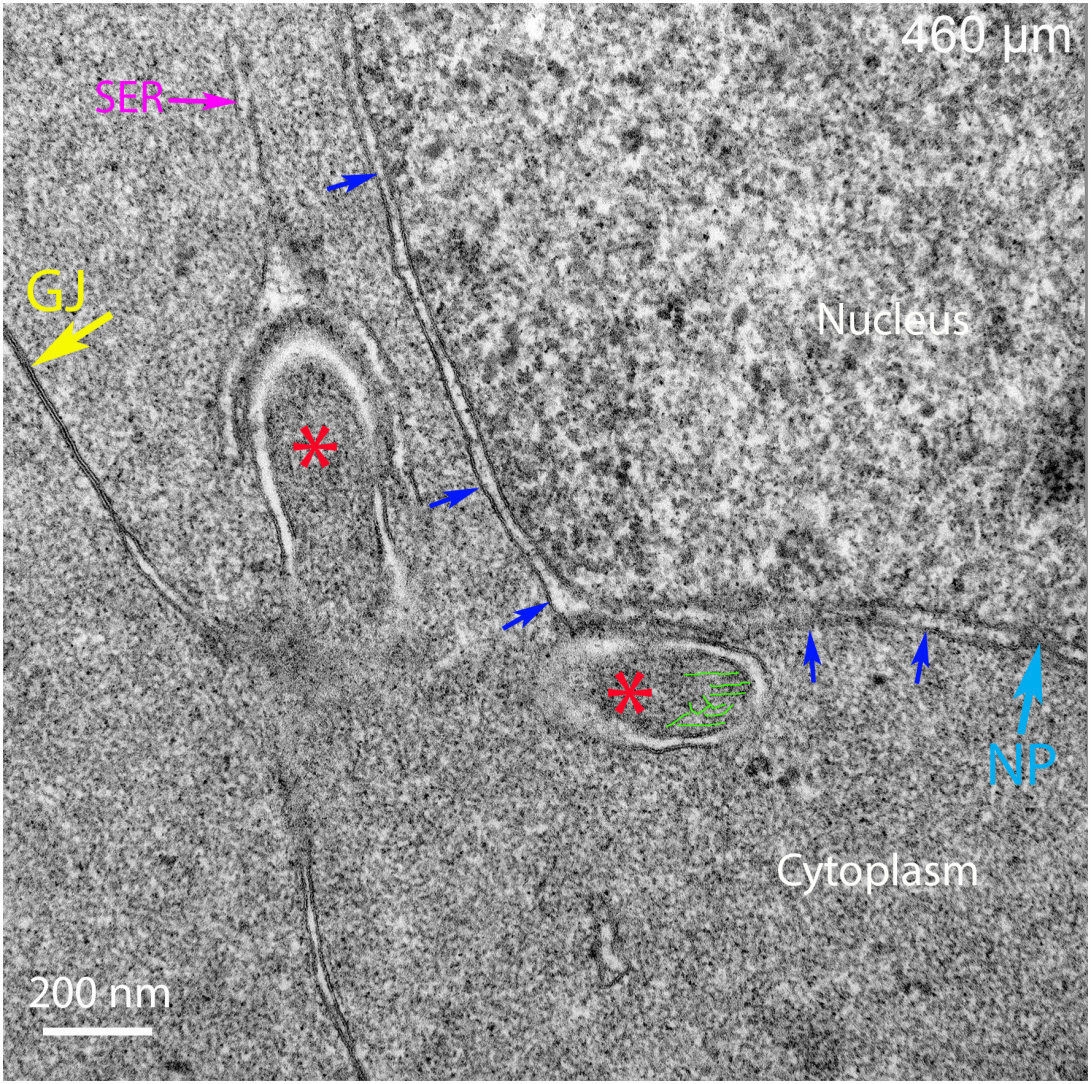
Extended structures appear to be early stages of complex formation. One extended structure is adherent to the outer nuclear membrane (blue arrows) with some internal microfilaments labeled (green lines) and potential core noted (asterisk). A second extended structure is capped by a single membrane organelle that is proposed to be smooth endoplasmic reticulum (SER). The needle-like projection and the apparent cap on the core are unique. An intact gap junction (GJ) is noted, as is a nuclear pore (NP). Day 15 lens about 460 μm from the capsule-fiber cell interface.

The finger-like projections noted at low magnification in the previous image (Fig 9A, black arrows) are clearly double membrane protrusions that tentatively are labeled as cores (Fig 10, asterisks), because they are uniform in density and one comes into contact with the outer membrane of the nuclear envelope (Fig 10), similar to the first example of a complex (Fig 6). The second finger-like structure is covered with a single membrane vesicle in an unusual configuration with a needle-like tip and possibly a cap over the projection (Fig 10). The origin and function of this structure is unknown, although the single membrane and clear cisternae suggest it may be a special form of smooth ER (Fig 10, SER). The base of these projections appears to originate from plasma membranes that are highly curved (not visible edge-on in the section) and blend into intact plasma membranes, which are continuous with a large gap junction (Fig 10, GJ). The geometry of the complex membrane interactions is not visible in this view, although other views provide a ready interpretation of the membrane inter-relationships.

**Fig 11:**
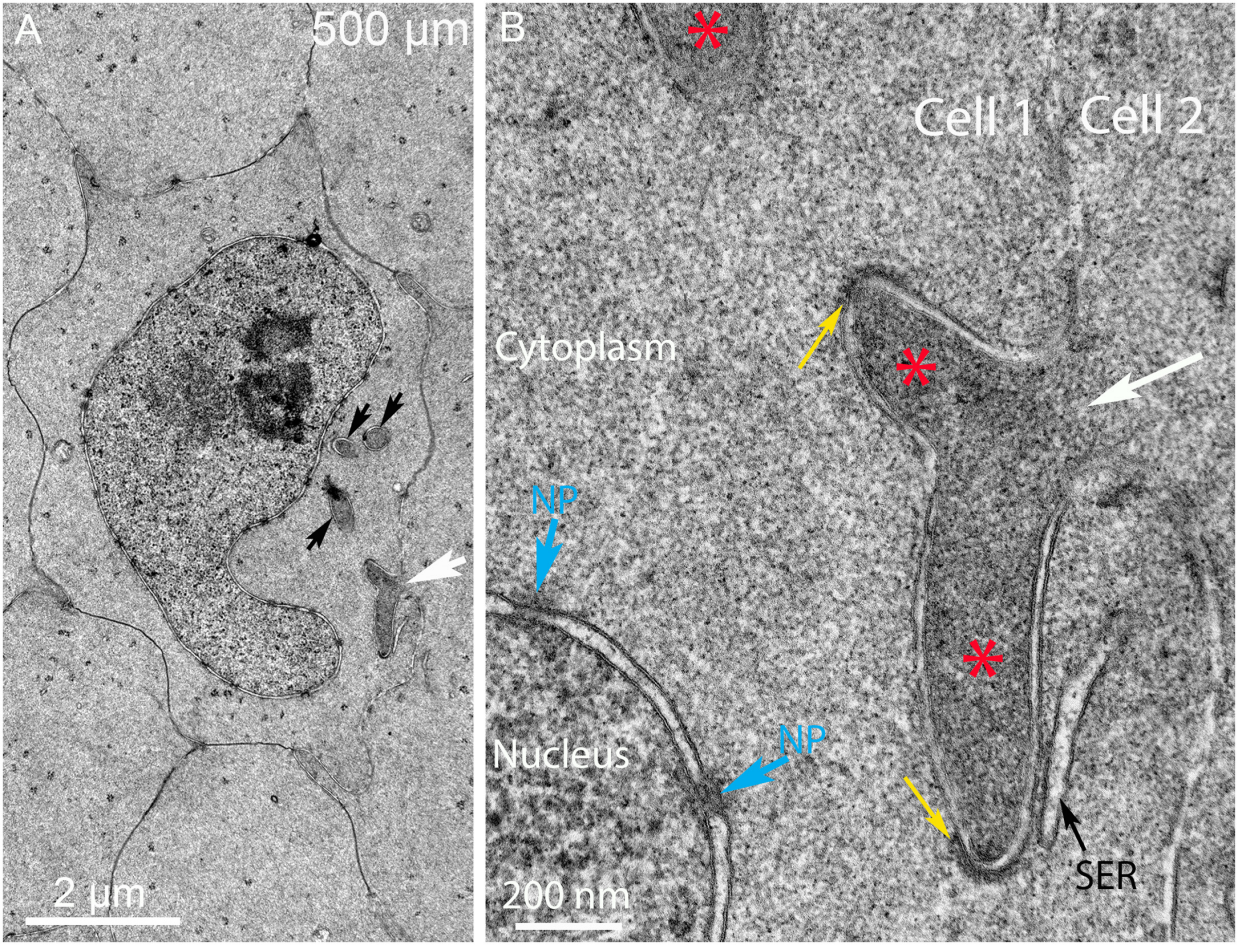
Degradation complexes originate as filopodial-like projections from adjacent cells. (A) Low magnification shows one cell sending a projection that bifurcates (white arrow) whereas three other profiles (black arrows) represent projections in different orientations and stages of development. (B) High magnification reveals that the projection from Cell 2 has two arms that have dark staining cores (asterisks) and well-defined clathrin-like caps (yellow arrows). One arm is closely associated with a single membrane flattened or tubular vesicle that appears to be smooth endoplasmic reticulum (SER). All of the cores are covered by double membranes confirming that they are derived from adjacent cells, as are many interlocking devices between fiber cells. Day 15 lens about 500 μm from the capsule-fiber cell interface.

Careful examination of the cellular structures around numerous nuclei visualized in this region of Day 15 lenses revealed extraordinary images of the early stages of complex formation initiated by projections from an adjacent cell (Fig 11A, white arrow). A reasonable interpretation of this image is that a projection initiated in Cell 2 bifurcates (Fig 11B), sending two similar projections into Cell 1 containing a nucleus in the critical stage of degradation. There are three additional projections cut at different angles also present (Fig 11A, black arrows), suggesting that, in general, the nucleus is attacked at multiple sites during the degradation process (also see S4 Fig). High magnification exposes several unusual features of this process (Fig 11B). First, the projection contains darkly stained cytoplasm and numerous filaments consistent with an actin network similar to the cores of the complexes described above (Fig 11B, asterisks; see S2 Fig for examples of proposed actin microfilaments). Second, the tips each have a clathrin-like coat (Fig 11B, yellow arrows). Third, the surface of one projection is in contact with a single membrane profile that appears to be smooth ER (Fig 11B, SER). These structures represent early stages of complex formation as the projections approach a region of nuclear envelope that appears to have intact nuclear pores (Fig 11B, NP). Although other interpretations of this image are possible, if this description is correct, then it is expected that projections with consistent properties will be found in different orientations.

**Fig 12:**
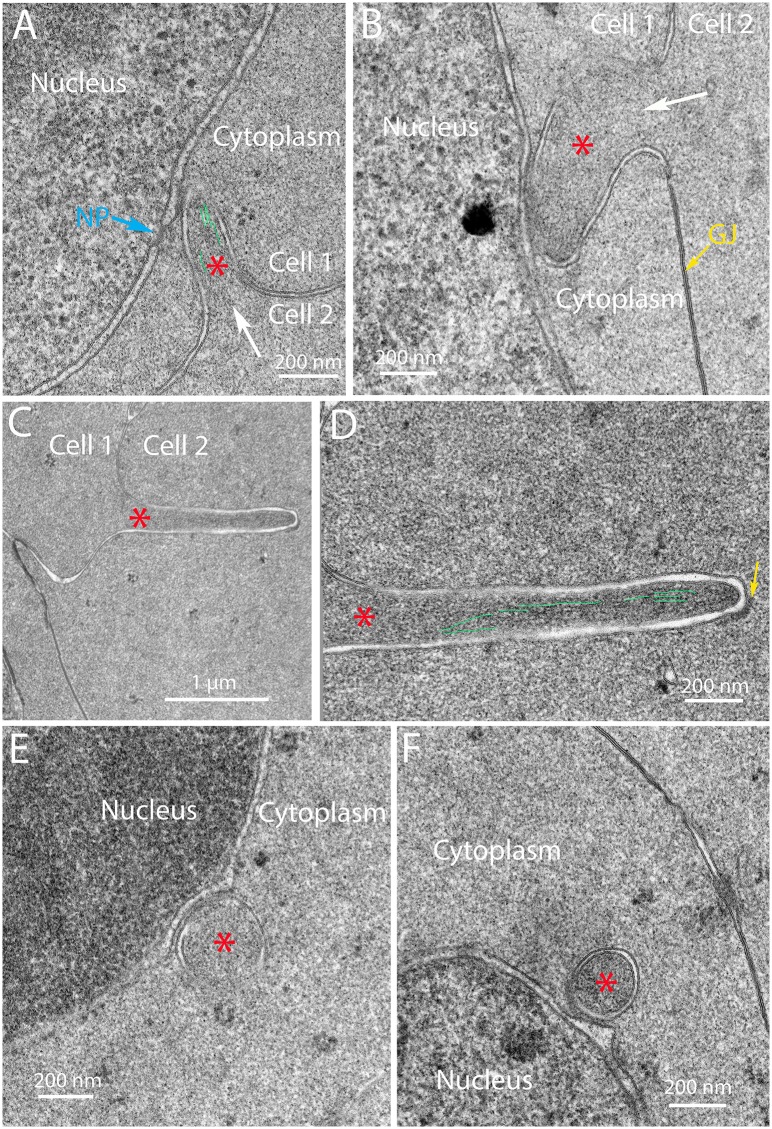
Different morphologies of filopodial-like projections support the hypothesis that they generate the degradation complexes. (A) and (B) Moderate magnification shows two projections with cores (asterisks) derived as projections from Cell 2 into Cell 1. Both are double membrane structures in which the outer membrane comes into contact with the outer nuclear membrane. Some internal microfilaments (green lines; also see S2 Fig) are visible and intact nuclear pores (NP) and gap junctions (GJ) are indicated. (C) and (D) Low and high magnification of an extended projection (also see S2 and S4 Figs) with internal microfilaments (green lines) and a clathrin-like cap showing that the projections are not simply ball-and-socket interdigitations, which are usually shorter, have a rounded bulging end and lose their clathrin coat at an early stage of formation [31]. (E) and (F) Circular double membrane profiles that are cross-sections of projections. Both examples have uniform cores (asterisks) and contact the outer nuclear membrane, with the projection making indentation in E and distorting the membrane into a triangular protrusion in F. Day 15 lenses about 600 μm from the capsule-fiber cell interface.

Several examples of projections are selected to further characterize these unusual cytoplasmic processes containing cores similar to those in the complexes described above (Fig 12, asterisks). Two examples show the direct attachment of the process from one cell to the nucleus in an adjacent cell (Fig 12A and 12B). Actin-like filaments are indicated in one core (Fig 12A) and a gap junction is indicated in another to confirm the boundary between adjacent fiber cells (Fig 12B). An example of a long projection that is not close to a nucleus illustrates that extensive process formation occurs within this region (Fig 12C and 12D; also see S2 and S4 Figs). These processes have some features similar to ball-and-sockets typical of mature fiber cells; however, this example clearly shows an important difference in that the projection has uniform width and does not bulge at the end. Moreover, a morphological study showed that ball-and-socket interdigitations were not present in chick embryo lenses [32], which can be confirmed by examining young fiber cells here (see Fig 5). At high magnification, the clathrin-like coat and internal actin-like filaments are indicated (Fig 12D). These projections seen in longitudinal section are easily distinguished from any organelles in this region near the OFZ. However, in cross-section, these projections appear like double-membrane vesicles that could easily be mistaken for autophagic vesicles (Fig 12E and 12F). These examples are clearly not autophagic vesicles, because they have uniform cores similar to the adjacent cytoplasm and are in contact with the outer membrane of the nuclear envelope, as in the first type of complex described (Fig 6). These images are consistent with the processes from adjacent cells attacking nuclei near the OFZ from numerous orientations and locations. Given this information, nearly all of membranous structures around nuclei in this critical region can be identified and none of the structures are consistent with the presence of lysosomes or autophagosomes at any stage. In the absence of interlocking devices, these images of filopodial-like processes at different orientations provide strong evidence that they are produced by adjacent cells to form the nuclear excisosomes and degrade fiber cell nuclear envelopes.

### F. Final stages of nuclear breakdown at the OFZ about 500–700 μm from the capsule

**Fig 13:**
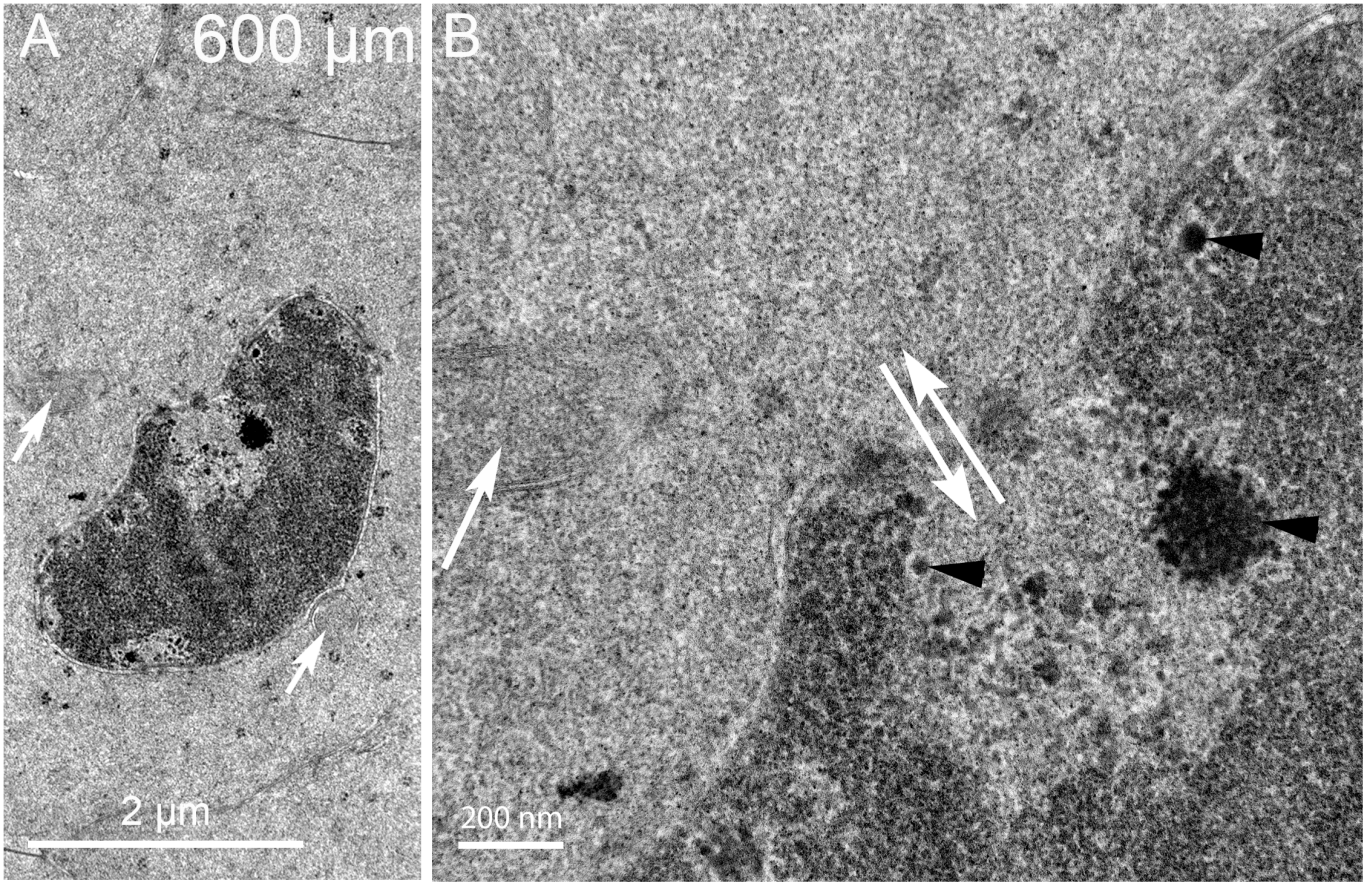
Nuclear envelope breakdown in a localized region reveals multiple staining patterns of the nucleoplasm. (A) Low magnification shows two projections near the nucleus (arrows) that exhibits several light staining regions as if material has been removed. (B) High magnification of a light-staining region shows that the nuclear envelope has been lost (double arrows) and that the nucleoplasm aggregates into small and large particles (arrowheads), some of which may have departed the nucleoplasm to create the lighter staining zones. This structure may represent a nucleus just prior to becoming pyknotic near the organelle-free zone. Day 15 lens about 600 μm from the capsule-fiber cell interface.

**Fig 14:**
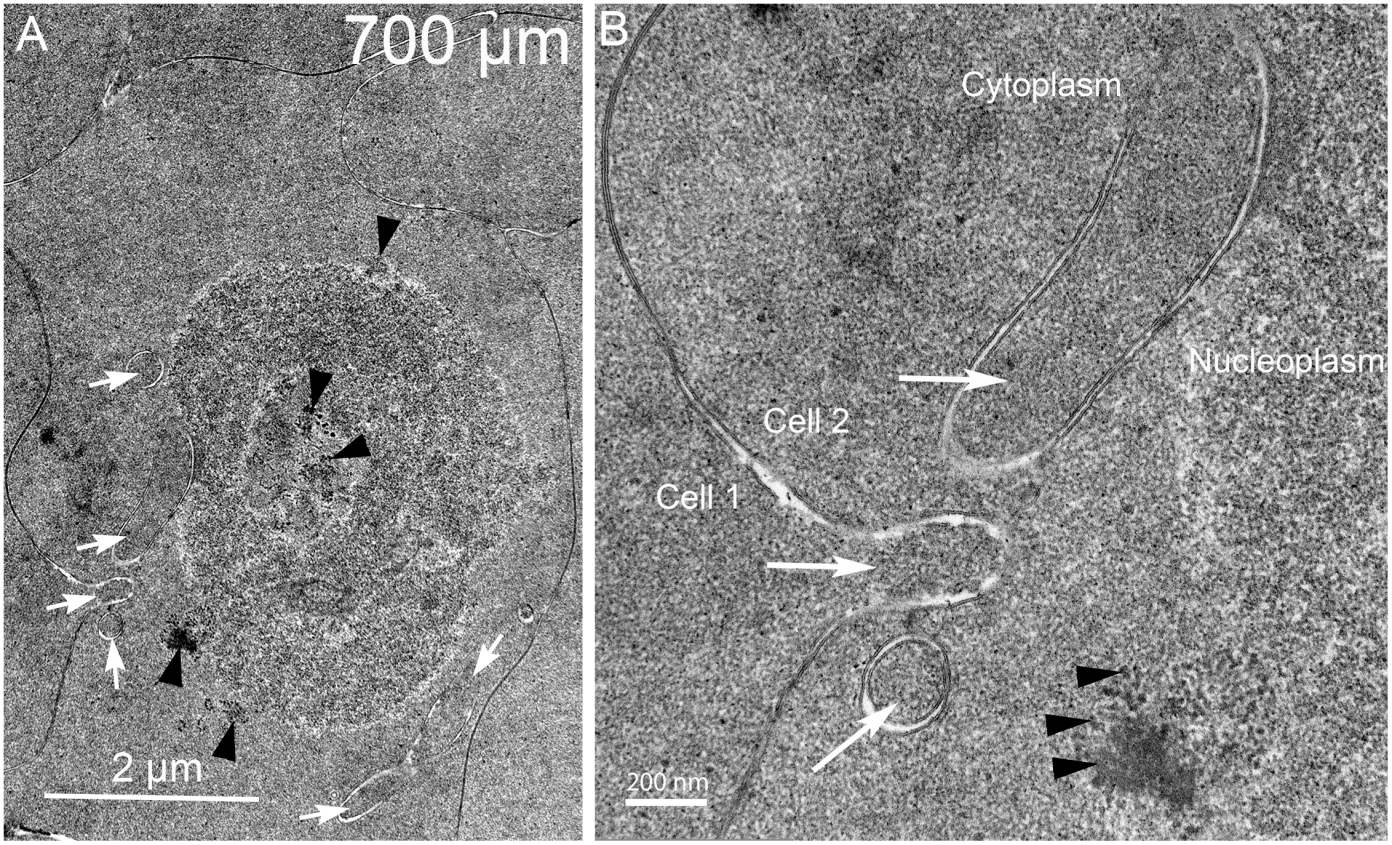
Nuclear envelope breakdown is accompanied by variable aggregations of remaining components of the nucleoplasm. (A) Low magnification shows multiple projections close to the nucleus (white arrows) even in the final stages of breakdown of the nuclear envelope. (B) High magnification near the center shows three projections (white arrows) adjacent to the nucleoplasm where there is no remaining nuclear envelope. The nucleoplasm displays different sized aggregates, some near 10–30 nm and others that are larger (arrowheads). Day 15 lens about 700 μm from the capsule-fiber cell interface.

Nuclei in Day 15 lenses at depths greater than 500 μm have variable appearances with some compromise or complete loss of the nuclear envelope. At low magnification, the nucleoplasm has been modified to have uneven density and enhanced granularity compared to earlier stages of degradation (Fig 13A). Also at low magnification, it is still evident that cytoplasmic processes are present around the nucleus (Fig 13A, white arrows). Enlargement of the central region shows that the nuclear envelope is no longer present in this location and that the nucleoplasm has been altered significantly (Fig 13B). The opening to a low-density region may represent a region of rapid exchange of components with the cytoplasm (Fig 13B, double arrows). This pattern may represent an advanced stage of degradation displaying a small nucleus with distinctive zones within the nucleoplasm and dense particles of varying size, which were not observed in young nuclei before degradation (Fig 13B, arrowheads). Overall, the granularity of the nucleoplasm is evident compared to the cytoplasm (Fig 13B). Another example of a late stage of nuclear degradation shows that the nuclear envelope has been completely removed, even though several filopodial-like processes are still present (Fig 14A, arrows). Within the core and at the edge of the nucleoplasm, several forms of condensation of nuclear components are noted (Fig 14A, arrowheads). The most common form is well-defined dense granules about 10–30 nm in diameter that are clustered and occasionally aggregated into a dark mass (Fig 14B), which is consistent with osmophilic deposits reported in early morphological studies [16, 18]. This pattern may represent a stage of final nuclear degradation. These two examples demonstrate that significant changes to the nucleoplasm occur simultaneously to degradation of the nuclear envelope and are further modified when the nucleoplasm is exposed to the cytoplasm. Similar degrading nuclei were observed in the confocal images (Fig 4C).

**Fig 15:**
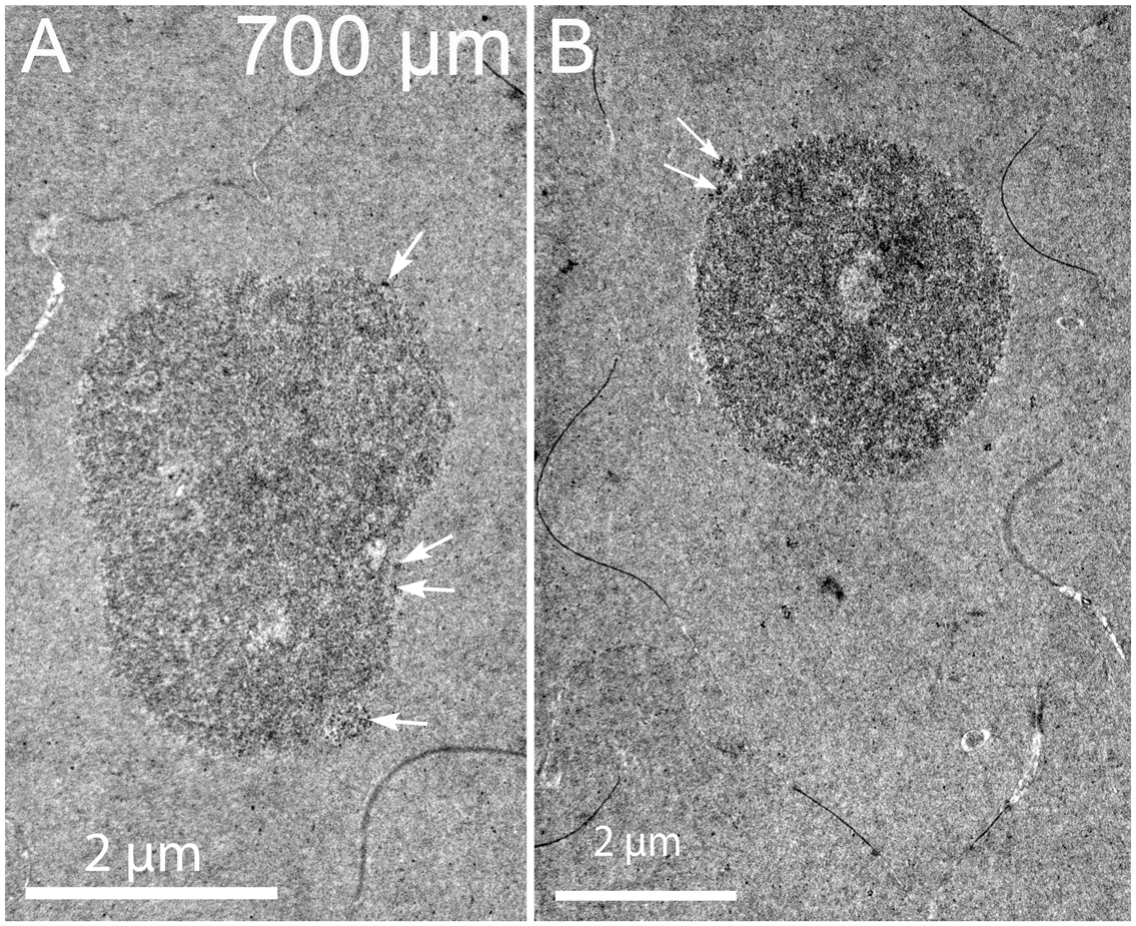
Nuclear remnants have many configurations without the nuclear envelope. A. and B. Two examples of globular remnants where no trace of the nuclear envelope is visible and yet the nucleoplasm forms micron sized aggregates composed of fine particles that have similar textures to the adjacent cytoplasm. Definitive structural data is not available to characterize the final stages of breakdown except to note that the larger aggregates appear to be shedding small dense particles around the surface (arrows). Note the absence of vesicles, intercellular processes and organelles, as well as the prominent irregular cell shapes in this region within the organelle-free zone. Day 15 lenses about 700 μm from the capsule-fiber cell interface.

Once the nuclear envelope is fully degraded, smaller fragments of the nucleus are observed, often with a fine granular appearance (Fig 15A and 15B). These may represent the final stages of nuclear degradation prior to absorption or breakdown of the remaining nuclear material, which has no apparent internal structure and is not associated with any membrane profiles. These low contrast materials may correspond to the smallest of the pyknotic nuclear fragments noted in earlier reports [9]. The disappearance of these nuclear remnants is the final stage in the formation of the organelle-free zone. As noted for the confocal images these forms still stain for double-stranded DNA (Fig 4D).

## Discussion

**Fig 16:**
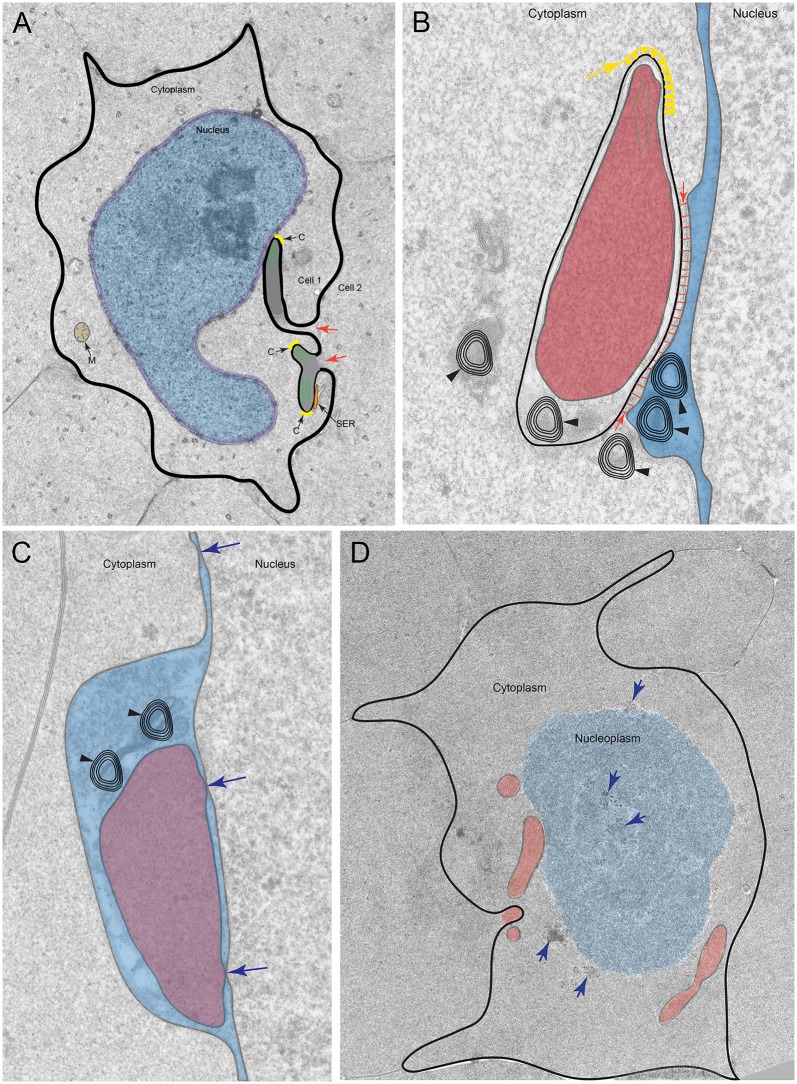
Diagram illustrating the steps in the formation of the nuclear excisosome and the breakdown of nuclei based on thin section electron micrographs. (A) Projections from adjacent cells approach an indented nucleus. Projections from Cell 2 into Cell 1 (red arrows) have a dense core containing an actin network (green lines) and a clathrin-like cap (C, yellow). A segment of smooth endoplasmic reticulum (SER, orange) is often seen adjacent to an initial projection. Mitochondria are usually present (M, tan). Based on the electron micrograph in Fig 11A with one process extended to contact the nuclear envelope. (B) Projections make contact with the outer nuclear envelope forming the initial nuclear degradation complex. The tip has a clathrin-like cap (yellow arrow) and actin microfilaments internally (green lines). The core (red) is similar to adjacent cytoplasm. The outer membrane of this double membrane complex appears to be anchored to the outer nuclear membrane (red lines and arrows) distorting the shape of the perinuclear cisternae (blue). Several multilamellar lipid-rich aggregates (black arrowheads) are located near the base of the complex. Based on the electron micrograph in Fig 6B. (C) Modifications of the complex allow close associations with the inner nuclear membrane to facilitate its degradation. Membrane rearrangements permit the core (red) to contact the nuclear envelope and be contained under the outer nuclear membrane within the perinuclear cisternae (blue). Multilamellar objects are also found within this space (black arrowheads). Nuclear pores (blue arrows) can be found within the complex. Based on electron micrograph in Fig 7B. (D) Remnants of the nucleoplasm, after the nuclear envelope has been degraded. Several processes (red) remain even in the absence of the nuclear envelope. Different types of aggregating components of the nucleoplasm (blue arrows) are observed. This type of remnant may represent a pyknotic nuclear fragment as reported in the early literature. Based on the electron micrograph in Fig 14A.

The ocular lens is an encapsulated stratified epithelial structure that grows throughout life by adding new layers of fiber cells derived from residual epithelial cells on the anterior surface. After the fiber cells produce the specific composition of proteins and membranes to support transparency, the lens completes its complex terminal differentiation program to degrade the organelles during formation of a transparent lens core. In the final stages of organelle degradation to form the OFZ, fiber cell nuclei are systematically removed employing a degradation complex that we discovered first attacks the outer leaflet of the nuclear envelope. Ultrastructural analysis of the degradation process in Day 15 chick embryonic lenses reveals the details of the formation of this unique complex, which we have called the nuclear excisosome, and exposes key steps in the degradation process (Fig 16). A unique feature of the nuclear excisosome is that each complex is composed, in part, of a filopodial-like projection from an adjacent cell that forms an intimate contact initially with the outer membrane of the nuclear envelope, thus bringing the inner surface of the plasma membrane into direct association with the nuclear envelope within each fiber cell at the critical stage of nuclear degradation. We are not aware of any other cellular organelle that supports such a contact in any vertebrate cell type. Furthermore, the nuclear excisosome is not observed in young cells outside the OFZ or in the core of lenses where organelle degradation is complete, suggesting that this is a unique organelle developed specifically by the vertebrate lens to degrade fiber cell nuclear envelope.

High resolution confocal microscopy has revealed brightly fluorescent objects associated with nuclear envelopes that are consistent with the TEM results (Figs 3 and 4). The lipid fluorescent dye stains plasma membranes that outline the fiber cells, the nuclear envelope membranes and membranous organelles. As a function of depth from the capsule, the internal cytoplasmic organelles decrease markedly as expected within the first 200 μm depth as new brightly staining oval or extended structures first appear at nuclear envelopes, which are most likely the nuclear excisosomes. By a depth of 300 μm, the background fluorescence from cytoplasmic components is so low that the predicted nuclear excisosome staining is prominent (Fig 4D). It is concluded that the high intensity of the fluorescence of the predicted nuclear excisosomes is most likely due to the aggregates of thin bilayer lipids recorded in the TEM images. Therefore, the lipid dye probably serves as a specific label for the nuclear excisosomes. The size of mature nuclear excisosomes is about 1–2 μm in confocal optical sections and has a similar extent in z-series (S1 Fig), which is close to the dimensions observed in TEM images (Figs 6–9). Fine cytoplasmic processes are faintly visible in confocal images (Fig 4A and S1 Fig) whereas they are the key features of nuclear excisosome formation in TEM images. The confocal images accurately record the degradation of nuclei leaving pyknotic nuclear remnants within the OFZ (Fig 4) as reported previously in early confocal studies [9].

Remarkably, during nuclear degradation, multiple filopodial-like projections from adjacent cells appear to be generated with clathrin-like coats and propelled by what looks like an internal actin network similar to that described in the formation of ball-and-socket interlocking devices [31]. These projections contact the outer nuclear envelope membrane and begin degradation by recovering lipid and degrading or displacing nuclear envelope proteins, as described in the diagram that shows distinct steps in the proposed degradation process derived from specific electron micrographs (Fig 16). The initial complex (Fig 16B) is hypothesized to fuse with the outer nuclear membrane to form a second configuration, which attacks the inner nuclear membrane (Fig 16C). Once the entire nuclear membrane is degraded, the nucleoplasm is exposed to cytoplasm and nuclear components aggregate in various patterns that are consistent with the final stages of nucleoplasm degradation. Simultaneous to the formation and action of the nuclear excisosome, it appears that the nucleoplasm is being modified and perhaps the total nuclear volume is reduced by an unknown mechanism as indicated by the nuclear indentations. These processes take place in prescribed locations depending on the stage of development. In Day 15 lenses examined, the process begins in the zone 100–300 μm from the CEI and ends at depths greater than 700 μm. In older embryos or adult lenses, the steps may be compressed into smaller radial distances as the OFZ enlarges with development and aging.

The primary enzyme thought to be responsible for degradation of nuclear chromatin is DNase IIβ [21], which was shown in mice to be necessary for denucleation because, in its absence in knock-out mouse lenses, the OFZ was not formed properly, causing cataracts due to undegraded nuclei [20]. DNase IIβ was shown to be present in lysosomes associated with nuclei near the OFZ and was thought to enter the nuclei and participate in degradation of the nucleoplasm [22]. Careful examination here of many nuclei in chick lenses during degradation did not reveal any lysosomes attached or fused with the nuclear envelope, although autophagosomes were identified in the vicinity of degrading nuclei along with other organelles including ER and mitochondria. The presence of lysosomes and DNase IIβ associated with the nuclear envelope in mice may be species specific, especially since the expression of this enzyme was transitory and localized to a few layers near the OFZ [22]. Recent studies have demonstrated that the nuclear lamina must be removed in mouse lenses by CDK1 (cyclin-dependent kinase 1) before DNase IIβ can enter the nucleus or before the nucleus can be degraded [25]. The proposed new function of CDK1 should be applicable to other species including chick lenses. Although direct visualization of the nuclear lamina in thin sections is difficult, a comparison of the inner and outer nuclear membranes in the region where nuclear degradation is in progress (300–500 μm deep) may be valuable. The membrane staining pattern is identical for inner and outer nuclear membranes at high magnification (see Figs 6B, 7B, 8B, 9B and 10) suggesting that the nuclear lamina adherent to the inner nuclear membrane may have been removed at an earlier stage of nuclear degradation. If DNase IIβ is shown to be present in chick lenses, it remains to be determined how it gains access to the nucleoplasm and how it becomes active within the nucleus.

Degradation of the nucleoplasm may be in part explained by the presence of many of the components of the ubiquitin-proteasome pathway in nuclei of differentiating fiber cells [23]. The evidence is compelling that key ubiquitin factors and processing proteasomes are present in fiber cell nuclei as they approach the OFZ [23]. Further evidence is provided that these components can also regulate the differentiation process during the removal of organelles including nuclei [24]. The hypothesis supported by this data suggests that this pathway influences the phosphorylation of lamin during the removal of the nuclear lamina as a prelude to the influx of DNase IIβ into the nucleus [24]. The combined activities of DNase IIβ and ubiquitin-proteasome pathway degradation could help explain the potential loss of nuclear volume indicated by the indentations before the nuclear envelope is finally degraded (in the zone 300–500 μm deep) and explain the final degradation of nuclear remnants (in the zone 500–700 μm and greater depth). Components, such as Ubc4/5, that are localized to the nuclear membrane [23] may participate in the processing of proteins released from the degradation of the nuclear membrane by the nuclear excisosome.

Comparisons to early ultrastructural studies of mouse lenses provide some insights about the degradation of the nucleoplasm and the sequence of events reported here [16, 18]. Two common features of these early studies were the presence of distinctive nucleoli with wheel-like densities and the movement of condensed chromatin degradation products through nuclear pores, which were not observed in chick lenses. Although these features may be species specific, it is possible that they signal the transition of the nucleus from protein production to degradation, which may also include modifications to the nuclear pores to allow release of partially degraded components within the nucleoplasm. If the nuclear pores are modified, then it may be possible that they can also provide access to the nucleoplasm for nucleolytic enzymes and ubiquitin-proteasome pathway components. The breakdown of the nuclear envelope was reported to be due to vesiculation [16] and primary lysosomes and multivesicular bodies were observed near the nuclear envelope [18], which were not observed in chick lenses as it is clear that the nuclear excisosome breaks down the nuclear envelope. In the absence of the nuclear envelope, the final stages of nuclear degradation reported in earlier studies produced products that were small 10–30 nm globular particles [33] and larger osmophilic densities [16, 18, 33], similar to objects observed in chick lenses (see Figs 13–15). Similar dense bodies were also observed in embryonic chick lens nuclei processed for TEM [17] suggesting that common pathways for disassembly of nuclear material and condensation of the degradation products occur before they are finally processed or absorbed into the cytoplasm to form the OFZ.

The interpretation of the confocal and TEM ultrastructural images provides important information about the mechanism of nuclear degradation in chick embryo lens fiber cells. First, the presence of an intact nuclear envelope at 300 μm depth (Fig 5) and its absence at 700 μm (Figs 14 and 15) suggest that we are observing the nuclear degradation process at the edge of the OFZ. Second, the only observed structure that consistently is found in contact with nuclear envelopes is the nuclear excisosome, as an extended structure in confocal images (Figs 3 and 4; S1 Fig) and in two forms in TEM images, one in contact with outer nuclear envelope (Figs 6, 10 and 12) and another in contact with the inner nuclear envelope (Figs 5–7; S3 Fig). Third, the contact regions of the nuclear excisosome show many examples of multilayered lipid aggregates which most likely are derived from the nuclear membranes as the membranes are being degraded. Fourth, the appearance of filopodial-like projections at the beginning of the degradation process (Fig 16A) and their continued presence as the nuclear envelope is fully degraded (Fig 16D) suggest that the entire process is influenced by these unique intercellular projections. Moreover, the absence of these projections in young newly formed fiber cells (Fig 5), as well as their absence in the lens core (Fig 15), supports this proposal. Additional experiments are needed to understand more fully the cellular components that regulate the association and proposed fusion of the nuclear excisosome with the outer nuclear membrane. It will also be important to provide further evidence by tomographic reconstructions or alternate microscopic methods of the direct contact of core structures with adjacent cells (as shown in Fig 12A and 12B). Separate studies are needed to characterize the parallel degradation of the nucleoplasm and whether the nuclear envelope is modified prior to the beginning of the degradation process described here. Experiments are underway to determine whether the nuclear excisosome is operating to degrade fiber cell nuclei in other species (Costello MJ, et al., Invest Ophthalmol Vis Sci. 2016;57:ARVO E-Abstract 3076) with the expectation that we are observing a general property of fiber cell differentiation.

## Materials and Methods

Research on chick embryos was performed using protocols approved by the Florida Atlantic University’s Institutional Animal Care and Use Committee (IACUC) and also follows United States Department of Agriculture and National Institutes of Health approved guidelines for research on chick embryos. Briefly, eggs were disinfected upon receipt and housed in an incubator. Prior to dissection, chick embryos were sacrificed by rapid decapitation to avoid potential pain. No embryos past day 15 were used in this study consistent with animal product guidelines that preclude the use of embryos three days or less prior to hatching.

Chick embryo lenses were obtained after timed maturation of the embryos from Day 12 through Day 15 (D12-D15). Fertilized White Leghorn chicken eggs were purchased as pathogen free (Charles River Laboratories, Storrs, CT) and were processed under sterile conditions at Florida Atlantic University. Embryos were euthanized by rapid decapitation at specific developmental stages following IACUC approved protocols. Lenses were dissected and immediately immersion fixed in 10% neutral buffered formalin in Boca Raton, FL (Florida Atlantic University) and shipped to Chapel Hill, NC (University of North Carolina) for final processing. After 24 hours formalin fixation, lenses (D12, n = 4, diameter = 2.0 mm; D13, n = 4, diameter = 2.1 mm; D14, n = 6, diameter = 2.2 mm; D15, n = 6, diameter = 2.3 mm) were fixed in 4% paraformaldehyde freshly prepared in 0.1 M cacodylate buffer for 48 hours as described previously [34]. Confocal specimens were prepared from Vibratome sections (160 μm thick) transferred to Tyrode’s buffer (Sigma-Aldrich, T2397), stained for membranes with DiI (ThermoFisher, Vybrant CM-DiI, V22888) for 15 minutes in 50% ethanol, washed twice for 5 minutes in buffer, followed by staining for nuclei with DAPI (Life Technologies, D1306) for 10 minutes followed by washing twice for 5 minutes in buffer. Stained sections were mounted with standard medium beneath a 17 μm thick glass coverslip. Confocal images were obtained with a Zeiss LSM 880 with Airyscan (Carl Zeiss Microscopy, Jena, Germany), using a Zeiss 63x oil numerical aperture 1.4 as the primary objective. Excitation for DiI and DAPI dyes was with diode lasers at 561 nm and 405 nm, respectively. The detector was the new Airyscan design that is essentially, using 32 pinhole detectors, able to increase resolution to 140 nm in the x,y-plane by capturing and analyzing more image data at the back focal plane of the objective. Resolution in the z-axis was also improved and in z-series stacks the thickness of each optical section was 200 nm. Two lenses from each age D12 to D15 were examined and all of the images presented are from the best preparation of a D15 Vibratome section.

Fixed lenses were stored in 0.1 M cacodylate buffer until processing for transmission electron microscopy as described previously [35]. Briefly, fixed lenses were Vibratome sectioned to 180 μm thickness, immersion fixed in 2.5% glutaraldehyde, 2% paraformaldehyde, 1% tannic acid in 0.1 M cacodylate buffer, pH 7.2 for 12 hours. After washing, fixed thick sections were stained in 0.5% osmium tetroxide at 4°C for 1 hour followed by en bloc staining in 2% uranyl acetate for 1 hour in 50% ethanol. Sections were dehydrated in an ethanol series, infused with propylene oxide and embedded in epoxy resin (Epon 812, EMS, Hatfield, PA). Thin sections 70 nm thick near the equatorial plane from the capsule to the lens center were cut with an ultramicrotome (Leica Ultracut UCT, Leica Microsystems, Vienna, Austria) using a Diatome diamond knife (Electron Microscopy Sciences, Hatfield, PA). Grids (hexagonal thin bar, 600 mesh) stained with uranyl acetate and lead citrate were examined for different stages of nuclear breakdown in an FEI T12 G2 transmission electron microscope (Hillsboro, OR) operated at 80 kV equipped with a high resolution CCD camera (Gatan model 794, Pleasanton, CA). Locations were imaged in relation to a typical Vibratome section of a D15 lens near the equatorial plane (Fig 1). Two lenses from each age were examined from about 40 thin section mesas. Similar structures were observed at each age after the OFZ started to form, although images from D15 lenses were emphasized for the manuscript. Measurements of microfilament diameter and thicknesses of membranes, junctions and thin bilayers were made on digital images at about 200,000x using a 4x loupe. The estimated precision is about ±0.2 nm. Measurements of membranes and bilayers were made only on regions in which they were oriented perpendicular to the image plane to give the smallest and most accurate dimension.

## Supporting Information

S1 Fig Confocal z-series at 350 μm depth. A Vibratome section stained with DAPI and DiI was optically sectioned in a z-series with 200 nm thickness for each section. Images were recorded on a Zeiss LSM 880 with the Airyscan detector in Super Resolution Mode giving an x,y-resolution of about 140 nm. Ten adjacent optical slices (1–10) are presented in which it is possible to follow five bright staining objects (magenta arrows) adherent to the nuclear envelope and three weaker staining strands (white arrows) that are potential links to the adjacent plasma membrane. Because most of the indicated structures are visible in 4–6 optical sections, they are roughly 1–1.5 μm in thickness along the z-axis.
(TIF)

S2 Fig Probable actin microfilaments in filopodial-like processes. Selected microfilaments from filopodial-like projections are all similar in diameter, roughly 7.5 ± 0.5 nm (n = 25). Lengths vary from 35 to 200 nm predominantly along the axis of the projections (red arrows). The average thickness of the plasma membrane and nuclear membranes are consistently about 7 nm and can serve as an internal standard. The average thickness of cytoplasmic actin filaments is about 6–8 nm depending on the cell type and method of preparation and they are easily distinguished from intermediate filaments (10 nm) and microtubules (24 nm). These microfilaments are indistinguishable from those seen in forming ball-and-sockets by direct imaging and confirmed with gold-antibody labeling [31]. (A) Image selected from Fig 6 representing an early stage of nuclear excisosome formation. (B) Image selected from Fig 11B representing an early stage of filopodial-like formation. (C) Image from Fig 10 showing that even short segments of filopodia have visible microfilaments. (D) Extended filopodial-like process from Fig 12. The prominent clathrin-like coat is indicated (arrow). (E) Extended filopodial projection in contact with a nucleus (N) and also displaying a clathrin-like coat (arrow). A low magnification view of this region is shown in S4 Fig. (F) Two filopodia near a nucleus visible at low magnification in S4 Fig.
(TIF)

S3 Fig Additional examples of nuclear excisosomes and thin bilayer multilamellar lipid aggregates. (A) A nuclear excisosome directly attached to the nuclear envelope where the contacts with the outer and inner nuclear membranes are clear (blue arrow and green arrow, respectively). The thin layers measure 5.1 nm (n = 16). An additional cluster of thin bilayers (arrowhead) is an example of their presence within the cytoplasm consistent with the hypothesis that the nuclear excisosome extracts lipid from the nuclear envelope and recycles it to local plasma membranes. Also see Fig 6. A projection from Cell 1 may be a component of the nuclear excisosome, which has many of its components out of the plane of section. (B) Thin lipid bilayer cluster in contact with plasma membranes (5.2 nm, n = 24). The presence of the gap junction (GJ) establishes that this cluster is not within the cytoplasm or part of a nuclear excisosome. Also see Fig 6. (C) A large spherical cluster of thin lipid bilayers (5.2 nm, n = 57) that has its outer layer continuous with the outer nuclear envelope (blue arrow) and rests in direct contact with the inner nuclear membrane (green arrow). The pattern of bilayers is significant because in addition to the 5 nm thickness, the high curvature in several locations and the point defect structures (white arrows) are typical of lipids but not of membranes containing proteins. (D) An early stage nuclear excisosome based on the few thin lipid bilayers; see also Fig 8. The contacts with the outer (blue arrow) and inner (green arrow) nuclear membranes suggest that this may be a site of lipid extraction from the nuclear envelope (by an unknown mechanism). In fact the multilamellar membranes vary in thickness 5–7 nm and can be compared with membranes from the nuclear envelope (7 nm), smooth endoplasmic reticulum (SER, 7 nm) and the gap junction (16 nm).
(TIF)

S4 Fig Filopodial-like projections are clearly visible in low magnification overviews. (A) Seven filopodial-like projections are indicated (arrows), three of which (5–7) are attacking the one nucleus and the others are attacking nuclei out of the field of view. The filopodia-like process 3 is shown at higher magnification in Fig 12 and S2 Fig. The thin lipid bilayer clusters at low magnification appear as dark plaques (arrowheads) shown here associated with the nuclear envelope and plasma membranes. (B) Three filopodia-like projections associated with an indented nucleus. Projection 1 is shown at high magnification in Fig 12F and 2 & 3 are shown in S2 Fig. Examples of thin bilayer clusters (arrowheads) are shown attached to the nuclear envelope and found within the cytoplasm. (C) A total of eight filopodial-like projections for these two nuclei is representative of the average of about four per nucleus. If each filopodial-like projection forms a nuclear excisosome, then each nucleus would be attacked at multiple locations simultaneously. Here projection 8 has a clearly visible clathrin-like coat at the contact site with the nucleus and contains microfilaments visible at high magnification in S2 Fig.
(TIF)
