# Supplementary figures and images for: Identification and Ultrastructural Characterization of a Novel Nuclear Degradation Complex in Differentiating Lens Fiber Cells
Source: PLoS One. 2016 Aug 18;11(8):e0160785. doi: 10.1371/journal.pone.0160785 (PMC4990417; doi:10.1371/journal.pone.0160785)

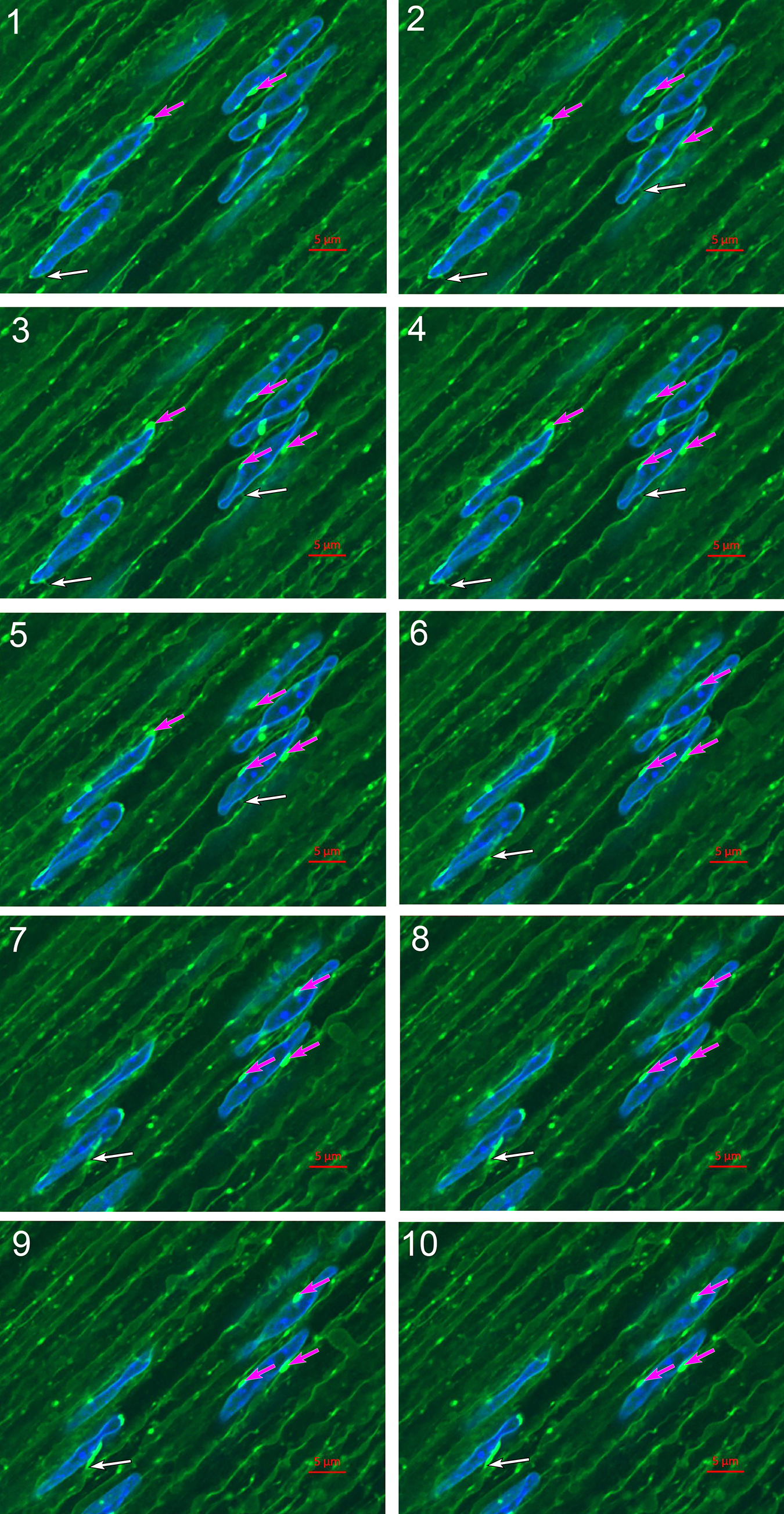

Supplement: S1 Fig — A Vibratome section stained with DAPI and DiI was optically sectioned in a z-series with 200 nm thickness for each section. Images were recorded on a Zeiss LSM 880 with the Airyscan detector in Super Resolution Mode giving an x,y-resolution of about 140 nm. Ten adjacent optical slices (1–10) are presented in which it is possible to follow five bright staining objects (magenta arrows) adherent to the nuclear envelope and three weaker staining strands (white arrows) that are potential links to the adjacent plasma membrane. Because most of the indicated structures are visible in 4–6 optical sections, they are roughly 1–1.5 μm in thickness along the z-axis. (TIF) [file pone.0160785.s001.tif]

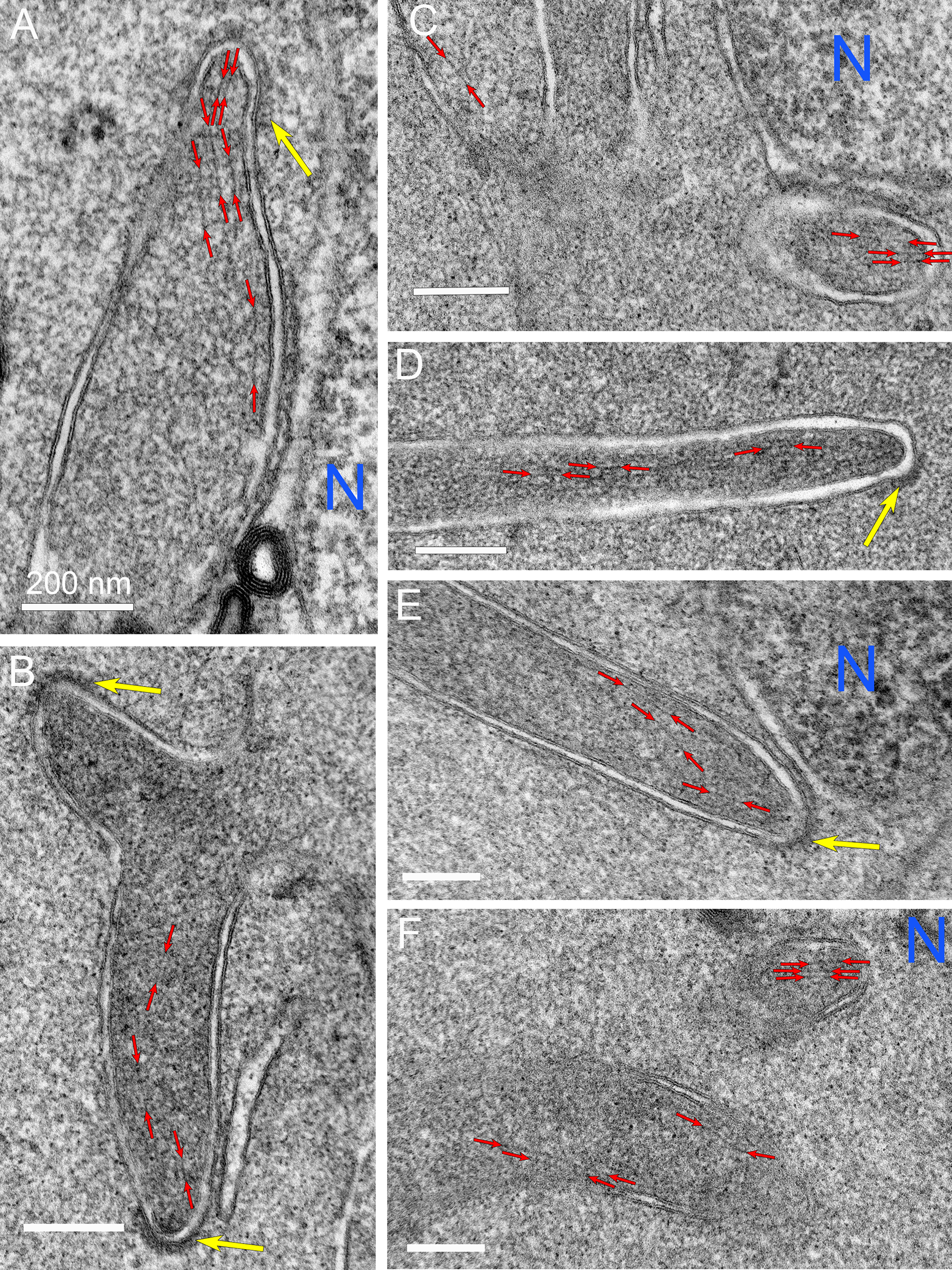

Supplement: S2 Fig — Selected microfilaments from filopodial-like projections are all similar in diameter, roughly 7.5 ± 0.5 nm (n = 25). Lengths vary from 35 to 200 nm predominantly along the axis of the projections (red arrows). The average thickness of the plasma membrane and nuclear membranes are consistently about 7 nm and can serve as an internal standard. The average thickness of cytoplasmic actin filaments is about 6–8 nm depending on the cell type and method of preparation and they are easily distinguished from intermediate filaments (10 nm) and microtubules (24 nm). These microfilaments are indistinguishable from those seen in forming ball-and-sockets by direct imaging and confirmed with gold-antibody labeling [31]. (A) Image selected from Fig 6 representing an early stage of nuclear excisosome formation. (B) Image selected from Fig 11B representing an early stage of filopodial-like formation. (C) Image from Fig 10 showing that even short segments of filopodia have visible microfilaments. (D) Extended filopodial-like process from Fig 12. The prominent clathrin-like coat is indicated (arrow). (E) Extended filopodial projection in contact with a nucleus (N) and also displaying a clathrin-like coat (arrow). A low magnification view of this region is shown in S4 Fig. (F) Two filopodia near a nucleus visible at low magnification in S4 Fig. (TIF) [file pone.0160785.s002.tif]

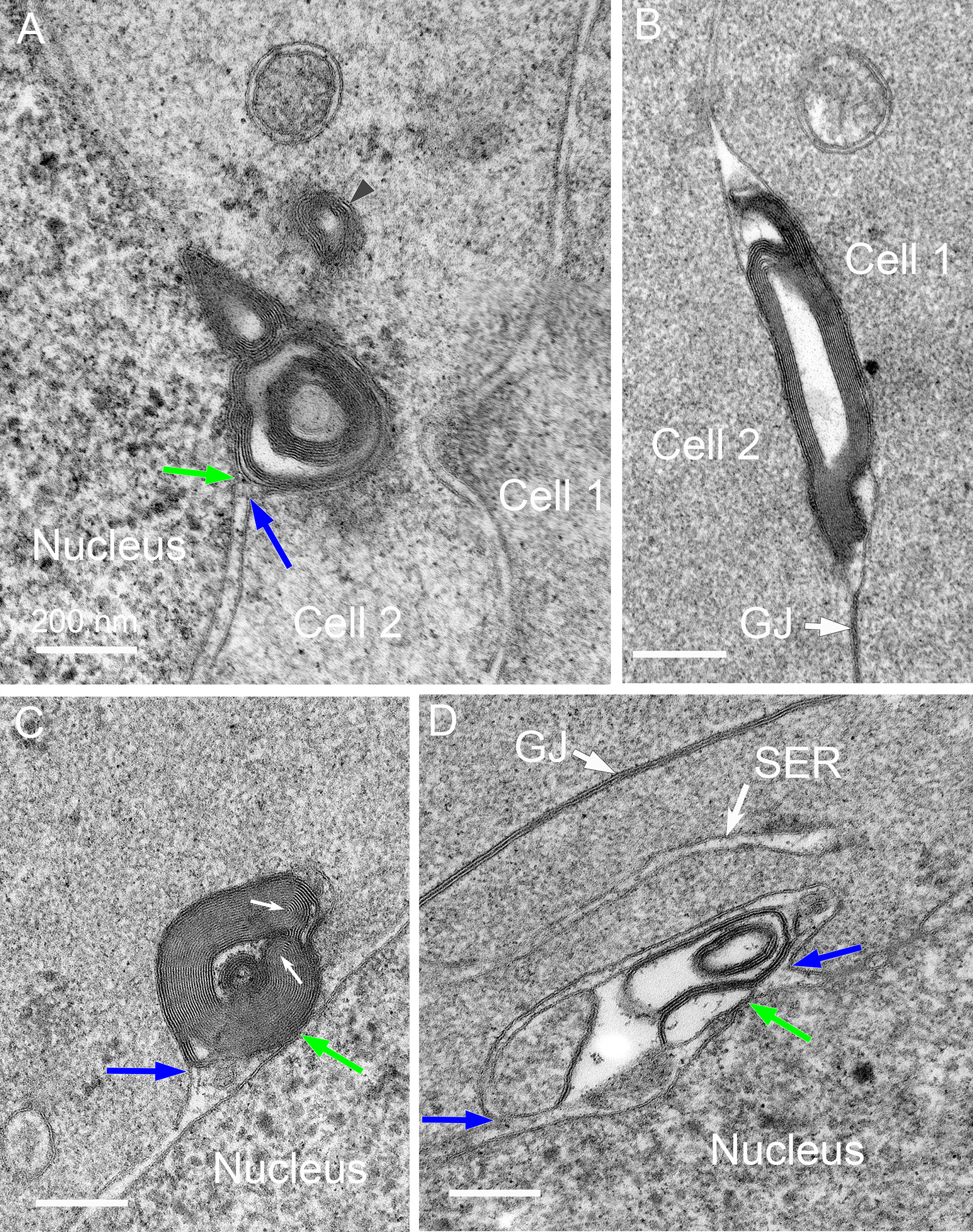

Supplement: S3 Fig — (A) A nuclear excisosome directly attached to the nuclear envelope where the contacts with the outer and inner nuclear membranes are clear (blue arrow and green arrow, respectively). The thin layers measure 5.1 nm (n = 16). An additional cluster of thin bilayers (arrowhead) is an example of their presence within the cytoplasm consistent with the hypothesis that the nuclear excisosome extracts lipid from the nuclear envelope and recycles it to local plasma membranes. Also see Fig 6. A projection from Cell 1 may be a component of the nuclear excisosome, which has many of its components out of the plane of section. (B) Thin lipid bilayer cluster in contact with plasma membranes (5.2 nm, n = 24). The presence of the gap junction (GJ) establishes that this cluster is not within the cytoplasm or part of a nuclear excisosome. Also see Fig 6. (C) A large spherical cluster of thin lipid bilayers (5.2 nm, n = 57) that has its outer layer continuous with the outer nuclear envelope (blue arrow) and rests in direct contact with the inner nuclear membrane (green arrow). The pattern of bilayers is significant because in addition to the 5 nm thickness, the high curvature in several locations and the point defect structures (white arrows) are typical of lipids but not of membranes containing proteins. (D) An early stage nuclear excisosome based on the few thin lipid bilayers; see also Fig 8. The contacts with the outer (blue arrow) and inner (green arrow) nuclear membranes suggest that this may be a site of lipid extraction from the nuclear envelope (by an unknown mechanism). In fact the multilamellar membranes vary in thickness 5–7 nm and can be compared with membranes from the nuclear envelope (7 nm), smooth endoplasmic reticulum (SER, 7 nm) and the gap junction (16 nm). (TIF) [file pone.0160785.s003.tif]

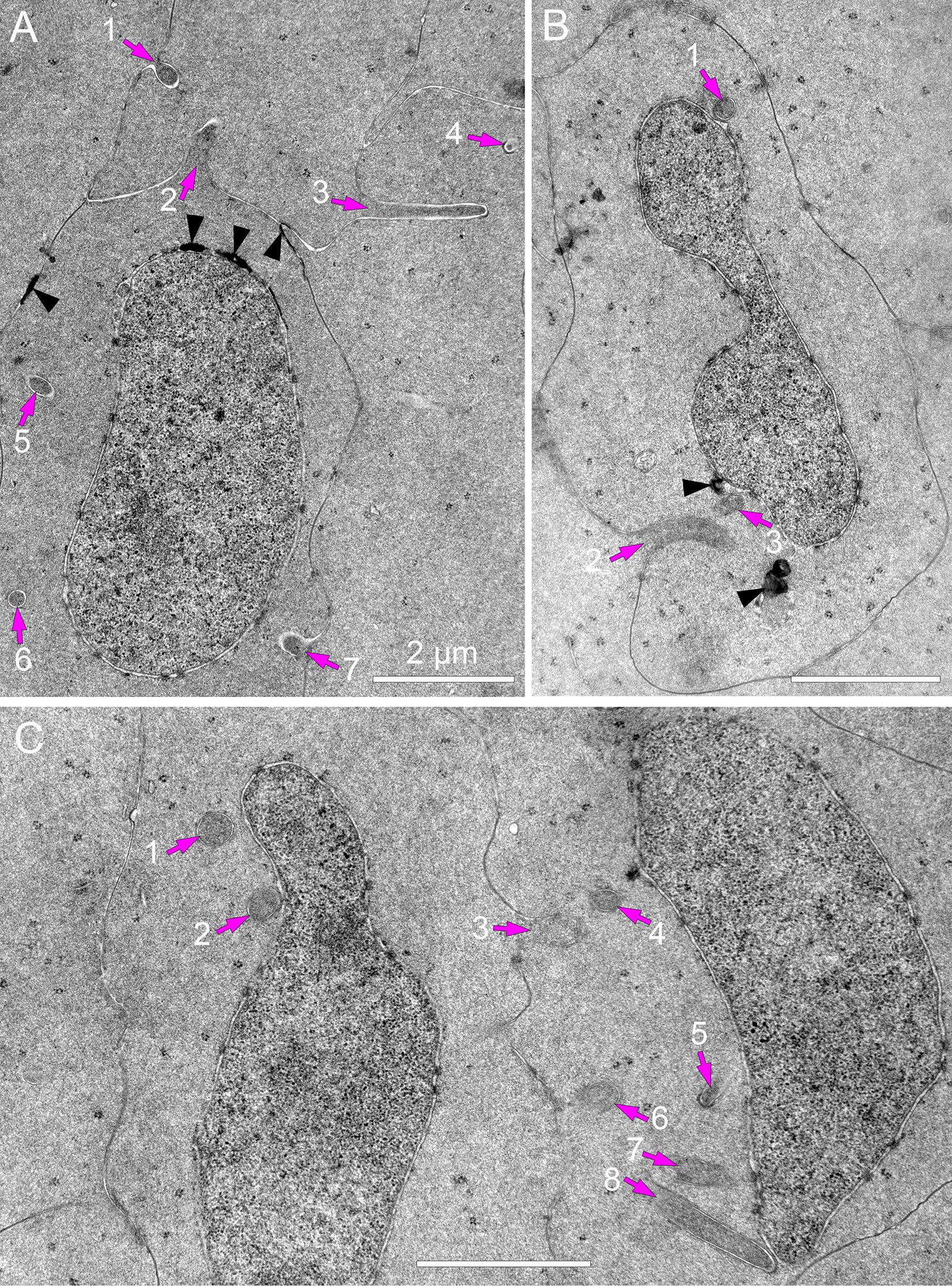

Supplement: S4 Fig — (A) Seven filopodial-like projections are indicated (arrows), three of which (5–7) are attacking the one nucleus and the others are attacking nuclei out of the field of view. The filopodia-like process 3 is shown at higher magnification in Fig 12 and S2 Fig. The thin lipid bilayer clusters at low magnification appear as dark plaques (arrowheads) shown here associated with the nuclear envelope and plasma membranes. (B) Three filopodia-like projections associated with an indented nucleus. Projection 1 is shown at high magnification in Fig 12F and 2 & 3 are shown in S2 Fig. Examples of thin bilayer clusters (arrowheads) are shown attached to the nuclear envelope and found within the cytoplasm. (C) A total of eight filopodial-like projections for these two nuclei is representative of the average of about four per nucleus. If each filopodial-like projection forms a nuclear excisosome, then each nucleus would be attacked at multiple locations simultaneously. Here projection 8 has a clearly visible clathrin-like coat at the contact site with the nucleus and contains microfilaments visible at high magnification in S2 Fig. (TIF) [file pone.0160785.s004.tif]
